# Circular RNA Tmcc1 improves astrocytic glutamate metabolism and spatial memory via NF-κB and CREB signaling in a bile duct ligation mouse model: transcriptional and cellular analyses

**DOI:** 10.1186/s12974-023-02806-w

**Published:** 2023-05-22

**Authors:** Danbi Jo, Yeong-Hwan Lim, Yoon Seok Jung, Young-Kook Kim, Juhyun Song

**Affiliations:** 1 Department of Anatomy, Chonnam National University Medical School, Seoyangro 264, Jeollanam-Do Hwasun, 58128 Republic of Korea; 2 Chonnam National University, Seoyangro 264, Hwasun, 58128 Republic of Korea; 3 Department of Biochemistry, Chonnam National University Medical School, Seoyangro 264, Hwasun, 58128 Republic of Korea

**Keywords:** Hepatic encephalopathy, Circular RNA (circRNA), Astrocyte, Bile duct ligation (BDL), NF-κB p65-CREB signaling

## Abstract

**Background:**

Hepatic encephalopathy-induced hyperammonemia alters astrocytic glutamate metabolism in the brain, which is involved in cognitive decline. To identify specific therapeutic strategies for the treatment of hepatic encephalopathy, various molecular signaling studies, such as non-coding RNA functional study, have been conducted. However, despite several reports of circular RNAs (circRNAs) in the brain, few studies of circRNAs in hepatic encephalopathy-induced neuropathophysiological diseases have been conducted.

**Methods:**

In this study, we performed RNA sequencing to identify whether the candidate circRNA cirTmcc1 is specifically expressed in the brain cortex in a bile duct ligation (BDL) mouse model of hepatic encephalopathy.

**Results:**

Based on transcriptional and cellular analysis, we investigated the circTmcc1-dysregulation-induced changes in the expression of several genes that are associated with intracellular metabolism and astrocyte function. We found that the circTmcc1 binds with the NF-κB p65-CREB transcriptional complex and regulates the expression of the astrocyte transporter EAAT2. Furthermore, circTmcc1 contributed to the secretion of proinflammatory mediators and glutamate metabolism in astrocytes and subsequently modulated an improvement in spatial memory by mediating neuronal synaptic plasticity.

**Conclusions:**

Thus, circTmcc1 may be a promising circRNA candidate for targeted interventions to prevent and treat the neuropathophysiological complications that occur due to hepatic encephalopathy.

**Supplementary Information:**

The online version contains supplementary material available at 10.1186/s12974-023-02806-w.

## Introduction

Hepatic encephalopathy (HE) is a neuropsychiatric syndrome that occurs in acute or chronic liver failure, and is accompanied by a wide range of neuropathological problems, such as cognitive impairment, ataxia, higher anxiety, personality change, depression, and coma [1–3]. The liver is the major organ that regulates lipid and glucose metabolism and eliminates toxic metabolites to ensure metabolic homeostasis in the body [4]. In disorders associated with hepatic failure, such as HE, dysfunctional liver detoxification causes excessive accumulation of toxic metabolites, such as ammonia, in the body and influences the central nervous system (CNS) function via circulation and crossing of the blood–brain barrier (BBB) circulation [5]. Toxic metabolites are transported to the brain, and ultimately result in neuronal cell damage and glial neuroinflammation that lead to cognitive impairment and depressive behavior [6]. Ammonia in the blood that is not metabolized due to liver dysfunction enters the brain and acts as a toxic substance [7]. In the CNS, astrocytes are the only cell type that removes excess ammonia, and this results in astrocyte swelling owing to ammonia excess that induces a severe inflammatory response and alteration of the glutamate–glutamine cycle, and eventually causes cognitive impairment [8–10]. Although there is significant evidence of the relationship between memory loss and liver dysfunction, the exact molecular astrocytic mechanisms that mediate this effect are unclear [11, 12]. Furthermore, previous mechanistic studies of the association between hepatic metabolic disorders and memory dysfunction have mainly investigated the function of protein-coding genes; few studies have evaluated the functions of non-coding RNAs (ncRNAs) [13, 14]. Among non-coding RNA types, circular RNAs (circRNA) constitute a type of ncRNA which is covalently closed by back splicing at the 5' to 3' ends of the exon–exon junction [15]. CircRNAs play roles in microRNA sponge, transcriptional regulators, and regulation of RNA-binding protein function [16]. Specifically, circRNAs are more abundant in the brain than in other tissues, and the transcriptional modulation of circRNAs could affect memory function by regulating neuronal differentiation and development as well as synaptic function [17, 18]. Recent studies presented the circRNA function in hepatic disorders and memory impairment, respectively. One study showed that circCREBBP inhibits liver fibrosis by binding to has-miR-129/LEFTY2 [18], whereas another revealed that circTUBD1 has been described as a regulator in radiation-induced liver fibrosis through binding to miR-203a-3p/Smad3[19]. Furthermore, in our previous studies, we demonstrated the roles of obesity-related circTshz2-2 in the regulation of memory function in the brain of obese mice [20] and the roles of non-coding RNAs in the regulation of neuronal cell death in the brain of mice with HE [21]. Nevertheless, no study has investigated the regulatory functions of specific circRNAs that are related to hepatic encephalopathy-induced cognitive decline. We hypothesized that the modulation of circTmcc1 may indicate a strategy to address neuropathological and neuropsychiatric issues in the HE brain.

We analyzed RNA sequencing data to identify a candidate circTmcc1 that was predicted to modulate neuropathological changes in the HE brain cortex. Furthermore, we verified whether circTmcc1 could regulate astrocyte function and thereby affect neural synaptic function and spatial memory ability in the HE brain.

## Materials and methods

### Cell culture

The mouse neuroblastoma Neuro-2A, mouse microglia BV2, and mouse astrocyte C8-D1a cell lines were purchased from American Type Culture Collection. The Neuro-2A cells (2 × 10^4^ cells per cm^2^) were cultured in minimum essential medium (MEM; WELGENE, Republic of Korea) with 10% fetal bovine serum (FBS; Millipore, USA), 100 U/mL penicillin–streptomycin (Thermo Fisher Scientific, USA), 1 mM sodium pyruvate (Thermo Fisher Scientific); the culture medium was replaced every 2 days. To induce neuronal differentiation of Neuro-2A, the culture media was replaced with Dulbecco′s modified Eagle’s medium (DMEM) with 2% FBS, and 100 U/mL penicillin–streptomycin, 1 mM sodium pyruvate, and 20 μM *all-trans* retinoic acid (RA, Sigma Aldrich) were added. The differentiation medium was replaced every 2 days.

The BV2 cells (2 × 10^4^ cells per cm^2^) were cultured in DMEM (WELGENE, Republic of Korea) with 5% FBS and 100 U/mL penicillin. The C8-D1a cells (2 × 10^4^ cells per cm^2^) were cultured in DMEM with 10% FBS and 100 U/mL penicillin; the medium was replaced every 2 days. To induce hyperammonemic conditions in cells, ammonium chloride (NH_4_Cl; Sigma Aldrich, USA) was diluted using sterilized water and the cells were treated with 20 mM NH_4_Cl for 24 h.

### Bile duct ligation surgery

Twelve-week-old male wild-type C57BL/6J mice (Koatech, Republic of Korea) were housed in the Laboratory Animal Research Center, Chonnam National University (CNU), under a 16-h light/8-h dark cycle at 23 °C with 60 ± 10% humidity and given ad libitum access to water and food when the experimental procedures were performed.

Mice underwent sham-operation or bile duct ligation (BDL) operation to induce the HE mouse model. Mice were anesthetized using 2,2,2-tribromoethanol/2-methyl-2-butanol (Sigma Aldrich) 0.2 mg/g (mouse body weight) via an intraperitoneal injection. Bile duct ligation was conducted using 5-0 silk suture under 1.5–2.0% isoflurane anesthesia (in a mixture of air and oxygen). Experiments were performed postoperatively for 2 weeks.

All experiments were conducted following the “96 Guidance for Animal Experiments” recommendation approved by the “Animal Ethics Committee” at CNU. The protocol was approved by the “Animal Ethics Committee” at CNU as CNU IACUC-H-2022-8.

### RNA-sequencing

Total RNA was prepared from the siRNA-transfected astrocyte cells using TRIzol Reagent (Ambion, USA) according to the manufacturer′s instructions. Deoxyribonuclease I (DNase I, Takara, Japan) treatment was used to remove the residual DNA. The isolated RNA was quantified using NanoPhotometer (Thermo Fisher Scientific). The Ribo-Zero rRNA depletion kit (Illumina, USA) was used to remove rRNA, and the TruSeq Stranded Total RNA Preparation Kit (Illumina) was used for the construction of total RNA-seq library that was applied to the NovaSeq 6000 System (Illumina).

The quality of the RNA sequencing reads was checked using FastQc, and the low-quality reads were trimmed by using the Trimmomatic algorithm [22]. Two different methods were used to analyze the specific expression change of circRNAs. In the first approach, STAR aligner was used for filtered reads to align with the mouse genome (mm10), and Cuffnorm was used for calculating the Fragments Per Kilobase of transcript per Million mapped reads (FPKM) [23, 24]. Genes with specifically altered expression were selected using the Student’s *t*-test. In the second approach, the quantification of the transcript level and calculation of differential expression was performed using a Salmon quantifier and edgeR, respectively [25]. By combining the results of the two approaches, the transcripts with a *p*-value of 0.05 or less were selected as the target in the analysis. In the RNA-seq results, genes with an average FPKM value less than 1 and 0 in any sample were excluded from the analysis. These genes were selected with a log2 value of more than 0.5 or less than − 0.5 of the differences in the expression changes between the control group and the experimental group.

### Bioinformatics analysis

Gene Ontology (GO) analysis was performed to analyze the function of the expression changed transcripts using Gene Ontology Resource (http://geneontology.org/). Differentially expressed protein-coding genes were analyzed using “biological process” in GO analysis. The biological process was analyzed using PANTHER overrepresentation and then tested using a type of Fisher’s exact test. The list has been sorted in the order of the false discovery rate (FDR) value as listed in Additional file 4: Table S3.

The BART (http://bartweb.org/) and ChEA3 (http://maayanlab.cloud/chea3/) tools were used to identify the transcriptional factors and chromatin regulators involved in circTmcc1-mediated EAAT2 regulation. A list of genes that were differently expressed by knockdown of circTmcc1 in RNA-seq data was entered via input into the BART and ChEA3 tool. Among the factors predicted in each tool, factors that were commonly included were selected. In BART and ChEA3, the top 10 factors were selected based on the Irwin–Hall *p*-value and Integrated Scale Rank, respectively. The transcriptional factors are listed in Additional file 4: Table S3.

The RPISeq (http://pridb.gdcb.iastate.edu/RPISeq/) tool was used to predict the interaction between circTmcc1 and transcriptional regulators. The sequence of circTmcc1 and the sequence of factors obtained from BART and ChEA3 were input into the RPISeq tool. The interaction probabilities were expressed as heat maps using the scores of random forest (RF) classifiers and support vector machine (SVM) classifiers. The scores of interaction probabilities are listed in Additional file 4: Table S3.

The STRING (http://string-db.org) tool was used to check the interaction network between transcription factors. The factors list was evaluated by the kmeans clustering and then classified into four clusters. This predicted network is listed in Fig. 4B.

### RNA isolation and PCR

Total RNA was isolated using TRIzol (Ambion) in accordance with the manufacturer's instructions. Complementary DNA (cDNA) was reverse-transcribed using a random hexamer, RevertAid reverse transcriptase (Thermo Fisher Scientific) from isolated RNA. A semi-quantitative PCR was conducted using Phusion High-Fidelity DNA polymerase (Thermo Fisher Scientific) in the Master cycler Nexus X2 (Eppendorf, Germany). PCR primers are listed in Additional file 2: Table S1.

### Subcellular fraction

Neuro-2A, BV2, and C8-D1a cells were fractionated into the nuclear and cytoplasmic parts. Cells were collected in PBS and treated with buffer A [10 mM HEPES buffer (pH 7.9), 10 mM KCl, 1 mM DTT and 0.1 mM EDTA (all products were purchased from Sigma Aldrich)]. Cells were incubated on ice for 25 min; then 10% Nonidet P-40 (NP-40) was added to cell suspensions for 2 min. After centrifugation of the cells, the supernatant containing the cytoplasmic fractions was separated using TRIzol LS reagent (Invitrogen, USA) to isolate the cytoplasmic RNA. The pellet was resuspended with K100 buffer D [20 mM Tris (pH 8.0, Thermo Scientific), 100 mM KCl and 0.2 mM EDTA] buffer and then centrifuged to obtain nuclear RNA by using TRIzol reagent. The precursor glyceraldehyde 3-phosphate dehydrogenase (pre-GAPDH) mRNA was used as controls of nuclear fraction, and mature GAPDH mRNA was used as the control for the cytoplasmic fraction.

### Western blot analysis

The cells and tissue were lysed in ice-cold radioimmunoprecipitation assay (RIPA) buffer (Translab, Republic of Korea) for 15 min on ice. The protein concentration of protein extract was quantified using a bicinchoninic acid (BCA) protein assay kit (Thermo Fisher Scientific). Protein (15 μg) was separated on 10–12% sodium dodecyl sulfate–polyacrylamide gel electrophoresis (SDS-PAGE), and the protein was transferred onto polyvinylidene fluoride membranes (PVDF; Millipore) activated by absolute methanol. The PVDF membrane was incubated with 5% bovine albumin (BSA, GenDEPOT, USA) and 5% skim milk (BD Bioscience, USA) in 1 × TBS-T buffer for 1 h and 30 min at room temperature. After incubation, the membranes were incubated with primary antibodies (1:1000 dilution) overnight at 4 °C: Primary antibodies: p-CREB (Cell Signaling Technology, 9198s), CREB (Cell Signaling Technology, 9104s), p-PKA (Santa Cruz Biotechnology, sc-293036), PKA (Santa Cruz Biotechnology, sc-376778), p-NF-κB p65 (Cell Signaling Technology, 3033s), NF-κB p65 (Abcam, ab16502), PSD-95 (Abcam, ab18258), SYP (Abcam, ab32127), EAAT2 (Santa Cruz Biotechnology, sc-365634), AQP4 (Santa Cruz Biotechnology, sc-32739), c-FOS (Santa Cruz Biotechnology, sc-166940), Gl syn (Santa Cruz Biotechnology, sc-74430), FZD6 (Santa Cruz Biotechnology, sc-393113), ADCY1 (Santa Cruz Biotechnology, sc-365350) and GAPDH (Santa Cruz Biotechnology, sc32233). After the primary antibody incubation, the membranes were incubated with horseradish peroxidase (HRP)-labeled secondary antibody (1:5000 dilution) for 2 h at room temperature. The membranes were visualized using an enhanced chemiluminescence (ECL) solution (Thermo Fisher Scientific) using Fusion Solo software (Vilber, France). Protein expression was analyzed using ImageJ (provided from NIH) and the protein level was normalized to the GAPDH protein level and the phosphorylation of protein was normalized to the total form of the protein.

### Cytokine array

C8-D1a conditioned media was used for the cytokine array (R&D Systems, USA) analysis according to the manufacturer’s instructions. The cell culture media was collected and centrifuged to obtain only the media. Cytokine array membranes were incubated in blocking solution (Array buffer 6) for 1 h at room temperature. After incubation, the blocking solution was removed and the membranes were incubated in sample mixture [supernatant + Array buffer 4] overnight at 4 ℃. The sample mixture was discarded and the membrane was incubated in Array buffer 4/6 containing detection antibody cocktail for 1 h at room temperature. Membranes were incubated in 1 × streptavidin–HRP solution for 30 min at room temperature, and then visualized using chemi-reagent mixture and Fusion Solo software. Dot blot was measured using ImageJ and the experimental dot blot was normalized to the control dot blot.

### Glutamine–glutamate detection assay

To measure the glutamine and glutamate level in astrocyte, glutamine and glutamate were detected using the Glutamine/Glutamate-Glo assay kit (Promega, USA) according to the manufacturer’s instructions. Astrocyte cell lysate was mixed with glutaminase enzyme solution (Glutaminase buffer, Glutaminase) and incubated for 40 min at room temperature. After incubation, the glutamate detection reagent was added, followed by 1 h incubation at room temperature. After incubation, luminescence of glutamine and glutamate were detected using GloMax Navigator (Promega).

### Ammonia detection assay

To measure the ammonia level in mouse plasma, ammonia was detected using ammonia assay kit (Abcam) according to the manufacturer’s instructions. Mice plasma was mixed with ammonia reaction mixture (Ammonia assay buffer, OxiRed probe, Developer, Enzyme mix and Converting enzyme) and incubated for 1 h at 37 ℃ in the dark. After incubation, the ammonia level was measured at 570 nm using an Epoch microplate reader (BioTeck, USA).

### Total and direct bilirubin detection assay

To measure the bilirubin (total and direct) level in mouse plasma, bilirubin was detected using the bilirubin colorimetric assay kit (BioVision, USA) according to the manufacturer’s instructions. Mice plasma mixed with total and direct bilirubin reagent mixture, respectively, were incubated for 30 min at room temperature. After incubation, the total and direct bilirubin probe solutions were added to each sample and the mixtures were incubated for 20 min at room temperature in the dark. Next, the total bilirubin level was measured at 600 nm and direct bilirubin level was measured at 550 nm using an Epoch microplate reader.

### ALT and AST detection assay

To measured alanine transaminase activity (ALT; Abcam) and aspartate amino aminotransferase (AST; Abcam) levels were measured in mouse plasma using each ELISA kit according to the manufacturer’s instructions. Mice plasma mixed with ALT antibody cocktail and AST antibody cocktail, respectively, and the plates were incubated for 1 h at room temperature. After incubation, TMB development solution was added and incubated for 10 min at room temperature in the dark. After incubation, stop solution was added and the ALT and AST levels were measured at 450 nm using an Epoch microplate reader.

### GABA detection assay

To measure gamma-aminobutyric acid (GABA) level in astrocyte, GABA concentration was detected using GABA ELISA kit (LS Bio, WA, USA) according to the manufacturer’s instructions. Astrocyte cell lysate was mixed with detection reagent A and incubated for 1 h at 37 ℃. After incubation, the detection reagent B was added, followed by 30 min incubation at 37 ℃. After incubation, TMB substrate solution was added and incubated for 10 min at 37 ℃. Finally, after incubation, stop solution was added and GABA concentration was detected at 450 nm using an Epoch microplate reader.

### cAMP detection assay

To measure cyclic adenosine monophosphate (cAMP) level in astrocyte, cAMP concentration was detected using cAMP ELISA kit (Elabscience, USA) according to the manufacturer’s instructions. Astrocyte cell lysate was mixed with biotinylated detection Ab solution and incubated for 45 min at 37 ℃. And then, after incubation, the HRP conjugate solution was added, followed by 30 min incubation at 37 ℃. After incubation, substrate reagent was added and incubated for 15 min at 37 ℃ in the dark. Finally, after incubation, stop solution was added and cAMP concentration was detected at 450 nm using an Epoch microplate reader.

### siRNA design and transfection

The siRNAs against the back-splicing junction of circTmcc1 were designed using the siDESIGN center on the horizon discovery (https://horizondiscovery.com/en/products/tools/siDESIGN-Center) and i-Score Designer (https://www.med.nagoya-u.ac.jp/neurogenetics/i_Score/i_score.html). The AccuTarget negative control siRNA and the circTmcc1 siRNAs were synthesized from Bioneer. The sequences of the siRNAs that were used in these experiments are listed in Additional file 3: Table S2.

For circTmcc1 knockdown, cells were transfected using Lipofectamine 3000 (Invitrogen) according to the manufacturer’s instructions. Cells were seeded (2 × 10^4^ cells per cm^2^) into multi-well plates and then siRNA-transfected at a final concentration of 30 nM.

### Circular RNA confirmation

To identify the circular form of circTmcc1, the RNA sample was mixed with RNase R (Epicenter) and 10 × RNase R buffer to remove linear RNA. The control sample was mixed with 10 × RNase R buffer only. RNase R digestion was conducted at 37 ℃ for 20 min and the reaction was inactivated at 95 ℃ for 5 min. The expression of the circular form of circTmcc1 is confirmed by synthesizing cDNA from the linear RNA-removed RNA sample.

### RNA-binding protein immunoprecipitation

RNA-binding protein immunoprecipitation (RNA-IP) was performed to identify the protein binding to circTmcc1. We used a Magna RIP kit (Millipore) according to the manufacturer’s instructions. Cells were collected in ice-cold PBS and then centrifuged. The separated cell pellet was lysed using RIP lysis buffer (RIP lysis buffer, protease inhibitor cocktail, RNase inhibitor) and incubated overnight at − 80 ℃. After incubation, the cell lysate was incubated with NF-κB p65 antibody-conjugated magnetic beads in RIP immunoprecipitation (IP) buffer (RIP wash buffer, 0.5 M EDTA, RNase inhibitor) for 48 h at 4 ℃ while rotating. The immunoprecipitate was mixed with proteinase K buffer (RIP wash buffer, 10% SDS, Proteinase K) and then incubated for 1 h at 55 ℃ while shaking. The RNA was isolated using phenol:chloroform:isoamyl alcohol (125:24:1, pH 4.3, Thermo Fisher Scientific) and chloroform (Thermo Fisher Scientific) were added and then precipitated in precipitate solution (Salt solution I, Salt solution II, Precipitate Enhancer, Absolute ethanol) overnight at − 80 ℃. The extracted RNA was reverse-transcribed to cDNA.

### Transient transfection and luciferase assay

The 293T (human embryonic kidney cells) cell line was obtained from the American Type Culture Collection (Manassas, VA, USA). All cell lines were maintained as described previously [26]. Mouse EAAT2 gene promoter (− 1 kb) was PCR-amplified from mouse genomic DNA (Promega, USA) using the primers (forward, 5′-CCTCCTTGTTTGAGGCACAG-3′ and reverse, 5′-CCCAAATGTCTGACCTGACC-3′) and inserted into the pGL3 basic vector (Promega) using the MluI and XhoI restriction enzyme sites. Transient transfections were performed using Lipofectamine 2000 transfection reagent (Invitrogen) according to the manufacturer’s instructions. Luciferase assay carried out as described previously [26]. Briefly, cells were transfected with indicated reporter plasmids together with expression vectors encoding p65 [27] and CREB [28]. An appropriate amount of empty vector was added to adjust the total DNA and siRNA (Additional file 2: Table S1) amounts used for each transfection to be 1 μg/well and then co-transfected with pCMV-β-gal plasmid as an internal control. Luciferase activity was measured at 48 h after transfection. The luciferase activity was normalized to b-galactosidase activity.

### Neurite length analysis

Neuro-2A cells (2 × 10^4^ cells per cm^2^) were seeded to measure the neurite length alteration by treating siRNA-transfected C8-D1a conditioned media. Conditioned media were treated in differentiated neurons and incubated for 24 h. After incubation, neurons were captured and analyzed using Image J to measure the neurite length. For neurite length analysis, more than 50 neurons per group were selected and analyzed in a blinded manner. The length of the longest axon among neurites extending from one soma was measured and compared. Neurite length data were normalized to siRNA control treated group data.

### Immunocytochemistry

C8-D1a cells (2 × 10^4^ cells per cm^2^) and Neuro-2A cells (2 × 10^4^ cells per cm^2^) were seeded on poly-d-lysine (PDL) coated cover slips in culture plates. Cells were fixed in 2% paraformaldehyde (Sigma) for 10 min and then incubated with primary AQP4, p-NF-κB p65, EAAT2, SYP and PSD-95 antibodies in gelatin detergent buffer (GDB) [0.1% gelatin (Sigma Aldrich), 0.3% Triton X-100 (Thermo Scientific), 16 mM sodium phosphate (Sigma Aldrich), 450 mM NaCl with pH 7.4 (Sigma Aldrich)] at 4 °C overnight. Cells were washed three times with 1 × PBS and incubated with secondary Alexa 488-conjugated anti-mouse IgG (Invitrogen) and Alexa 594-conjugated anti-rabbit IgG (Invitrogen) antibodies for one hour at room temperature. Cells were counterstained using mounting medium with 4′,6′-diamidino-2-phenylindole (DAPI, Thermo Scientific). Cells were visualized using a K1-Fluo confocal microscope (Nanoscope systems) and fluorescence intensity were analyzed using ImageJ software.

### Osmotic pump implantation

The osmotic pump was filled with a 100 μL siRNA-siPORTNeoFX solution (Sterile saline, siPORTNEOFX, siRNA for target RNA) at a final concentration of 5 μM. A siRNA-filled osmotic pump and brain infusion kit were assembled and incubated in sterile saline overnight at 37 ℃.

The sham and BDL mice were anesthetized using 0.2 mg/g 2, 2, 2-tribromoethanol/2-methyl-2-butanol and maintained in 1.5–2.0% isoflurane anesthesia (in a mixture of air and oxygen). The osmotic pump-brain infusion kit assembly were infused into the lateral ventricle of mice (mediolateral 1.0 mm, anteroposterior 0.3 mm based on the bregma as a landmark) using stereotaxic instrument (Harvard Apparatus, USA) for 3 days.

### T-maze behavior test

The T-maze behavioral test was performed using white plastic T-maze apparatus with three arms (35 × 9 × 15 cm, length × width × height). The test was conducted in accordance with a previously reported protocol, but with slight modifications, to assess cognitive ability [29]. The mice were adapted to the apparatus for 10 min before the initial trial. The test was conducted with the mice placed on the back of the start arm and allowed the apparatus to move freely for 10 min. The movement of mice was recorded using a digital camera and each recorded video was converted into image sequences. These image sequences were used for mouse movement track line using plugin, the Animal Tracker of ImageJ. The total distance, velocity, and immobility time data of the mice were extracted from the parameters of locomotor activity. The percentage of spontaneous alternation was calculated as: [(number of alternations)/(total number of arm entries − 2) × 100%].

### Statistical analysis

All data are presented as the group mean ± S.E.M. Statistical analysis was conducted using unpaired two-tailed *t*-test with Welch's correction in Prism 8 (GraphPad Software Inc, USA). Data were considered significant at *p < 0.05, **p < 0.01, and ***p < 0.005 in the statistical analysis.

## Results

### Transcriptomic analysis of brain cortices in the bile duct ligation (BDL) mice model

**Fig. 1:**
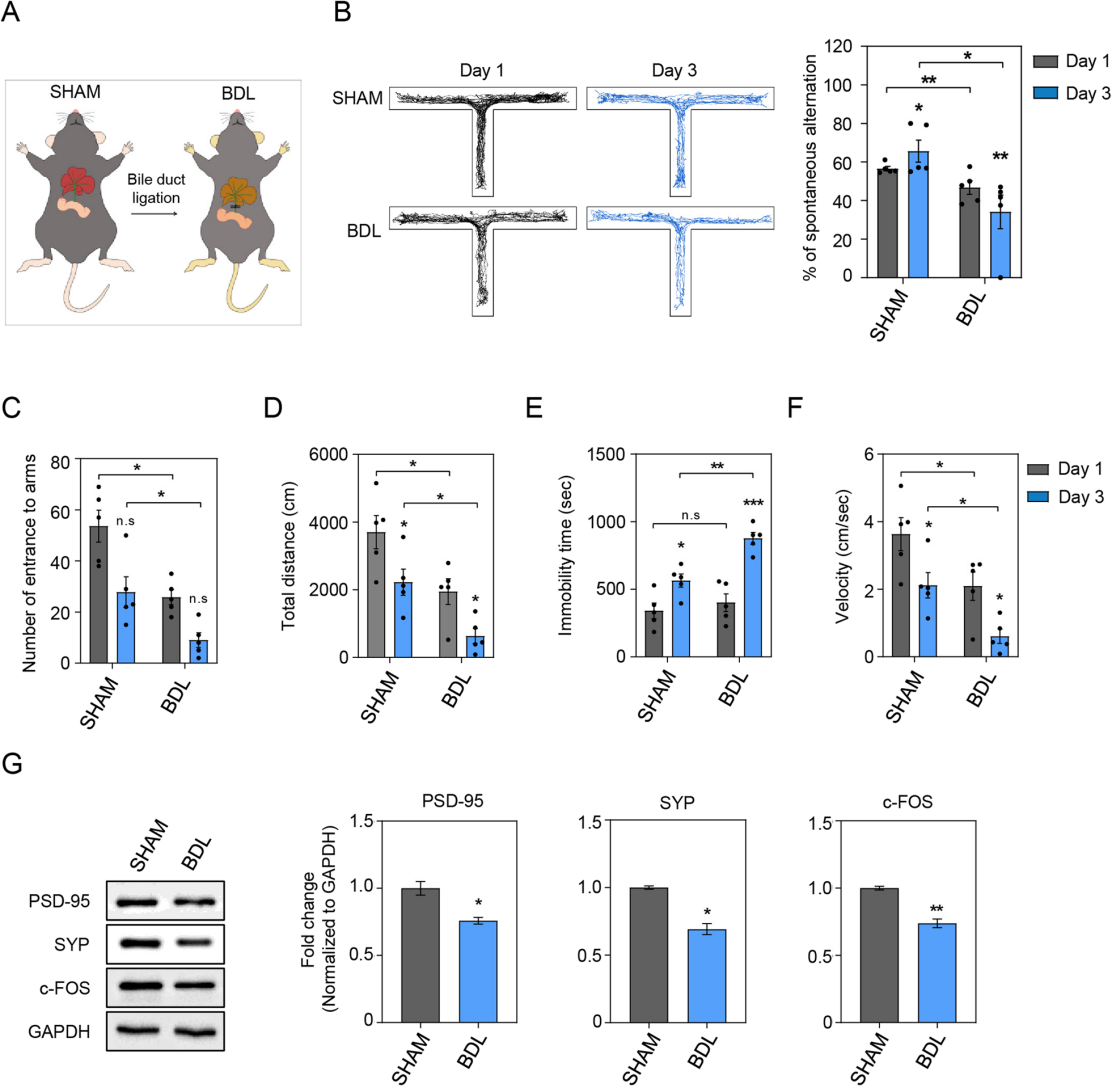
The suppression of synaptic plasticity and cognitive function in the BDL mouse brain cortex. **A** Illustration displaying the procedure of RNA sequencing in the BDL mice cortex tissues. **B** The movement of sham and BDL mice in T-maze were represented by illustration. The track line of movement indicates 10 min of measurement for five mice per group. In sham and MDL mice, the change in percentage of spontaneous alternation was expressed as mean ± SEM (*n* = 5) and shows the percentage of spontaneous alternation of T-maze test on days 1 and 3. In **C**–**F**, data were measured at days 1 and 3 during the experiment. **C** The number of entrances to arms, **D** the total length of mouse movement (cm), **E** the immobility time of mouse (seconds), and **F** the velocity of mouse movement (cm/s), for sham and BDL mice, respectively. Data were measured at days 1 and 3 during trials and were reported as the mean ± SEM (*n* = 3). **G** The measurement of PSD-95, SYP, and c-FOS protein levels in the sham and BDL mouse brain cortex. Data are represented as mean ± SEM (*n* = 3). An unpaired two-tailed *t*-test with Welch’s correction was used for statistical analysis. ns, not significant, *p < 0.05, **p < 0.01, ***p < 0.001

In this study, we used the bile duct ligation (BDL) surgery mouse model [30, 31] which is a common HE mouse model that is used to investigate the brain functions in hepatic encephalopathy (Fig. 1A). Firstly, at 2 weeks after BDL surgery, we resected brain cortices in the BDL mouse brain and also measured liver function-related markers in the plasma to test whether the liver function of BDL mice was impaired. We found that BDL surgery increased AST and ALT levels more than three times compared to that in sham mice (Additional file 1: Fig. S1A) [32]. Increased level of AST and ALT two enzymes in BDL mouse blood plasma indicates damage to the hepatocytes [33]. Bilirubin, as one of the components of bile acid, is made from hemoglobin breakdown in blood and is considered as a main index to evaluate liver dysfunction [34]. Compared to sham mice, the levels of total bilirubin and direct bilirubin in BDL mice were more than three times higher, which suggests that bilirubin was not excreted due to the ligation of the bile duct (Additional file 1: Fig. S1B). The ammonia level in the blood of BDL mice was measured using an ELISA kit, and was dramatically increased compared to those of sham mice (Additional file 1: Fig. S1C). Although the preoperative body weight of sham and BDL mice was similar (27.4 ± 1 g vs. 27.5 ± 0.3 g in sham and BDL groups, respectively), the postoperative body weight significantly decreased in the BDL group 13 days after BDL surgery as compared to the sham group (27.5 ± 1 g vs. 19.4 ± 1 g in sham and BDL groups, respectively; Additional file 1: Fig. S1D). Based on the detection of these liver function-related markers, we inferred that BDL mice had severe liver failure and hyperammonemia.

Next, we performed total RNA sequencing analysis using the brain cortices in sham and BDL mice to check specific transcriptomic changes in the BDL mouse brain [21]. After RNA sequencing transcriptome analysis, we selected eight circRNAs among 23 upregulated circRNAs and 24 downregulated circRNAs that were differentially expressed in BDL mouse cortex; circTmem44, circRere, circCdyl, circTmcc1, circMkln1, circZzz3, circTtc3, circCrebrf (Additional file 3: Table S2). Among circRNAs that showed significantly higher fold-changes, we selected circTmcc1 in the BDL mouse cortex (Additional file 1: Fig. S2).

### Evaluation of impaired spatial memory and synaptic dysfunction caused by BDL surgery

To assess whether BDL surgery contributes to cognitive dysfunction, we measured the alteration of spatial memory in BDL mice using the T-maze behavior test (Fig. 1B–F). Spontaneous alternation of the T-maze tests was based on the willingness of rodents to prefer exploring a new environment [29]. To evaluate the spatial memory ability, the movement and locomotor activities such as number of entrances to arms, total distance, immobility time, and velocity were measured and analyzed in the T-maze test (Fig. 1B–F). We observed a spatial memory impairment in BDL mice compared to sham mice based on the T-maze test scores. The spatial memory capacity in the sham mice had improved on Day 3 compared to that on Day 1 of the test. However, the spatial memory capacity in the BDL mice was significantly decreased on Day 3, suggesting that the spatial learning function is related to liver dysfunction. We assume that these data indicate not only spatial memory impairment, but also decreased locomotor activities in BDL mice as compared to sham mice (Fig. 1B–F).

To measure whether BDL surgery affects synaptic function in BDL mouse (Fig. 1G), we checked the protein expressions of both presynaptic (synaptophysin, SYP) and postsynaptic (postsynaptic density protein-95, PSD-95) markers using Western blotting analysis. We observed that the protein levels of these markers were reduced in the BDL mouse brain compared with that in sham mice [35]. In addition, we found decreased protein levels of c-Fos, a neuronal activity marker, in the BDL mouse brain [36].

### Identification of circTmcc1 expression in BDL mouse brain

**Fig. 2:**
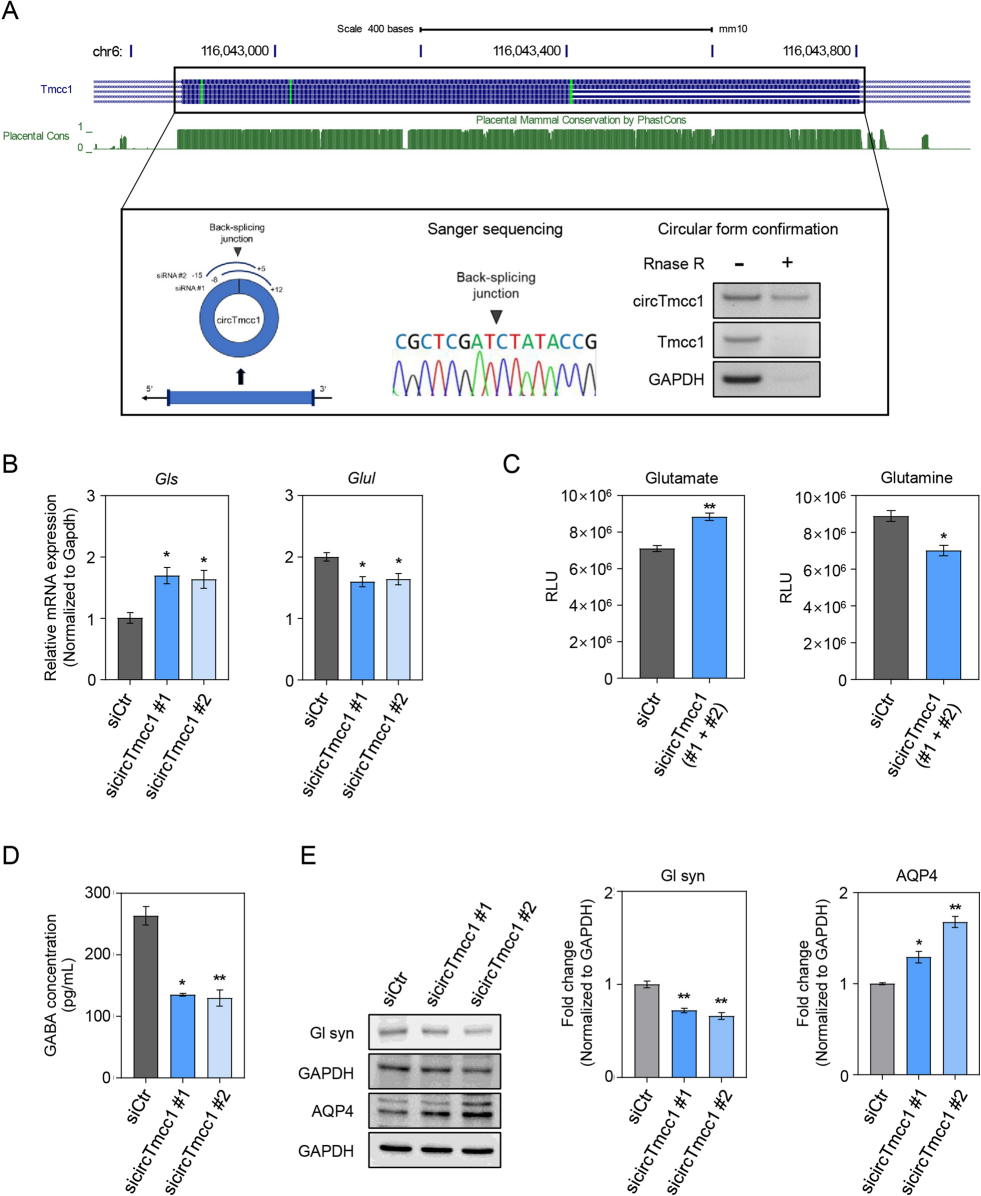
circTmcc1 regulates astrocytic function in BDL mice cortex. **A** Genomic information of circTmcc1 in mouse Tmcc1 locus (mm10). This information and PhastCons data were obtained from the UCSC Genome Browser. The siRNAs were designed to bind to the back-splicing junction of circTmcc1. Confirmation of the circular formation of circTmcc1 with RNase R treatment. **B** Comparison of mRNA expression of glutamate and glutamine synthesis-related genes (Gls, Glul) in circTmcc1-downregulated C8-D1a cells. The data are reported as mean ± SEM (*n* = 3). **C** Measurement of intracellular glutamate and glutamine levels in C8-D1a cells after circTmcc1 knockdown. The data are expressed as three independent experiments in duplicate. **D** Measurement of GABA concentration in C8-D1a cells after circTmcc1 knockdown. The data are expressed as three independent experiments in duplicate. **E** The measurement of the expression of Gl syn and AQP-4 protein in circTmcc1-downregulated C8-D1a cells. The expression changes are presented as the mean ± SEM (*n* = 3). An unpaired two-tailed *t*-test with Welch’s correction was used for statistical analysis. ns, not significant, *p < 0.05, **p < 0.01

Firstly, to investigate the functional roles of circRNAs in BDL mouse brain, we determined the expression level of circTmcc1 according to the CNS cell types, such as neuron (Neuro-2A), microglia (BV2), and astrocyte (C8-D1a; Additional file 1: Fig. S3A). This confirmed that circTmcc1 and Tmcc1 are expressed sequentially in neuron, astrocyte, and microglia (Additional file 1: Fig. S3A). We checked the genomic locus of the transcript of circTmcc1 using UCSC Genome Browser identifiers, which is composed of one of the exons of Tmcc1. We used sanger sequencing analysis to identify the junction site of circTmcc1 generated from the back-splicing of exons (Fig. 2A). To confirm whether circTmcc1 has a circular structure, the total RNA of each cell line was treated with RNase R to remove linear RNAs. However, circTmcc1 expression in each cell line was unaffected by RNase R treatment and was observed at the expected size (Fig. 2A; Additional file 1: Fig. S3B, C). We found that, unlike circTmcc1, the linear RNAs Tmcc1 and *Gapdh* were degraded by RNase R treatment in each cell line.

No study of the function of circTmcc1 has been conducted; however, it has been reported that Tmcc1 regulates ER recruitment [37]. Therefore, the tissue distribution of Tmcc1 was confirmed using data from the public GTEx portal (http://gtexportal.org/home/). The human *TMCC1* gene is expressed in various tissues and is specifically expressed in diverse brain regions, including the cerebellar hemisphere, cerebellum, cortex, and hippocampus (Additional file 1: Fig. S4). The expression of circTmcc1 and the Tmcc1 mRNA were confirmed in the brain cortical, hippocampal, and striatal tissues (Additional file 1: Fig. S5A–C). The expression of circTmcc1 was reduced in cortical tissues, whereas the expression of Tmcc1 did not change in all of the tissues that were evaluated (Additional file 1: Fig. S5A–C). CircTmcc1 and Tmcc1 were expressed not only in brain tissue, but also in lung, liver, muscle, fat, spleen, gut, and kidney (Additional file 1: Fig. S5D).

### circTmcc1 regulates the expression of glutamate transport in astrocytes under hyperammonemic conditions

We performed subcellular fractionations to identify the intracellular distributions of circTmcc1 in each cell type. CircTmcc1 was mainly distributed in the cytoplasm of each cell, whereas Tmcc1 is distributed equally in the nucleus and the cytoplasm (Additional file 1: Fig. S6A). These results indicate that circTmcc1 might modulate cellular functions in the cytoplasm. To investigate the function of circTmcc1 in the HE brain, ammonium chloride (NH_4_Cl) treatment of cells under hyperammonemic conditions [38, 39] considered as the in vitro mimicking of the HE-related condition. The expression of circTmcc1 in cells exposed to ammonia was minimally changed in Neuro-2A neuronal cell line and decreased in the BV2 microglia cell line and the C8-D1a astrocyte cell line (Additional file 1: Fig. S6B). These in vitro results are similar to that of the expression pattern of circTmcc1 in the in vivo BDL mouse cortices.

To confirm the functional roles of circTmcc1, we designed two types of small-interfering RNAs (siRNAs) for silencing the expression of circTmcc1 by binding to the back-splicing junction of circTmcc1. Moreover, knockdown using two types of siRNA was performed for 48 h in cells under hyperammonemic toxic conditions. Cells were transfected with independent siRNAs using the FUGENE reagent. Downregulation (~ 50%) of circTmcc1 indicates little change in Tmcc1 host gene expression (Additional file 1: Fig. S6C). Additionally, we determined whether the inhibition of circTmcc1 expression in hyperammonemic conditions could affect the cellular function in each cell. To identify the function of circTmcc1 in neuronal cells, we measured the changes of neuronal complexity and the alteration of neuronal function-related genes. In hyperammonemia-treated Neuro-2A cells, inhibition of circTmcc1 expression did not affect the neuronal complexity including neurite length, number of secondary branches, and number of neurites from soma compared to the control siRNA-treated Neuro-2A cells (Additional file 1: Fig. S7A). We measured the mRNA levels of neuronal differentiation-related genes Rbfox3, biosynthesis of the neurotransmitter-related genes Chat, microtubule-related genes Map2, and presynaptic function-related genes Syp in differentiated Neuro-2A cells under hyperammonemic conditions [40, 41]. The knockdown of circTmcc1 in Neuro-2A cells induced no significant difference in the expression of neuronal function-related genes (Additional file 1: Fig. S7B). These results indicate that the inhibition of circTmcc1 did not even minimally affect the neuronal complexity and neuronal function of neuronal cells directly. Next, we checked whether circTmcc1 regulates the inflammatory response in microglia under hyperammonemic conditions. In BV2 microglia cell, circTmcc1 did not contribute to changes in the expression of proinflammatory cytokine-related genes (*Tnf-α*, *IL-6*, *IL-1β*, *Ptgs2*, and *Mcp1*) and anti-inflammatory cytokine-related genes (*IL-10* and *Tgf-β*; Additional file 1: Fig. S7C) [42]. Suppression of circTmcc1 does not affect the inflammatory response in microglia that are exposed to the hyperammonemic environment. To check whether circTmcc1 is associated with the glutamate metabolism of astrocyte, we measured the mRNA levels of the expression of glutaminase (gls) which is a amidohydrolase enzyme that converts glutamine to glutamate [43], and *glul* is a glutamine synthetase that converts glutamate to glutamine and ammonia (Fig. 2B) [44]. Inhibition of circTmcc1 increased mRNA level of *gls* and decreased the expression of *glul* (Fig. 2B). Moreover, we measured the glutamate/glutamine level of intracellular concentration with the astrocyte glutamine/glutamate-glo assay kit (Fig. 2C). The concentration of intracellular glutamate in astrocyte increased with the suppression of circTmcc1, whereas the concentration of glutamine decreased (Fig. 2C). These results suggest that circTmcc1 is involved in excessive accumulation of glutamate and its conversion to glutamine in astrocytes. To confirm that circTmcc1 regulates the concentration of gamma-aminobutyric acid (GABA), a neurotransmitter, we measured intracellular GABA concentration in astrocytes using a GABA ELISA kit (Fig. 2D). The concentration of intracellular GABA in astrocyte decreased according to the suppression of circTmcc1 (Fig. 2D). These results indicate that circTmcc1 depletion induces dysregulation of glutamate metabolism in the glutamate–glutamine cycle. To confirm the function of circTmcc1 in the C8-D1a astrocyte cell line, the protein expression of Glutamine synthetase (Gl-syn) and aquaporin-4 (AQP4) as a transmembrane water channel were measured using Western blotting (Fig. 2E) [45]. We observed that the expression of Gl-syn decreased and AQP4 in astrocytes increased, owing to the inhibition of circTmcc1 (Fig. 2E). Moreover, we detected astrocyte swelling in circTmcc1-depleted astrocytes. The C8-D1a astrocyte was transfected with circTmcc1 siRNAs and control siRNA and stained with DAPI for identifying the nucleus, and with an anti-AQP4 antibody for the water channel of astrocyte. The fluorescence intensity of AQP4 was higher in the circTmcc1-depleted astrocytes than in the control siRNA-treated cells (Additional file 1: Fig. S7D). These results suggest that circTmcc1 regulates astrocyte activation and glutamate metabolism under hyperammonemic conditions.

### circTmcc1 alters the expression of genes related to intracellular metabolism in astrocytes

**Fig. 3:**
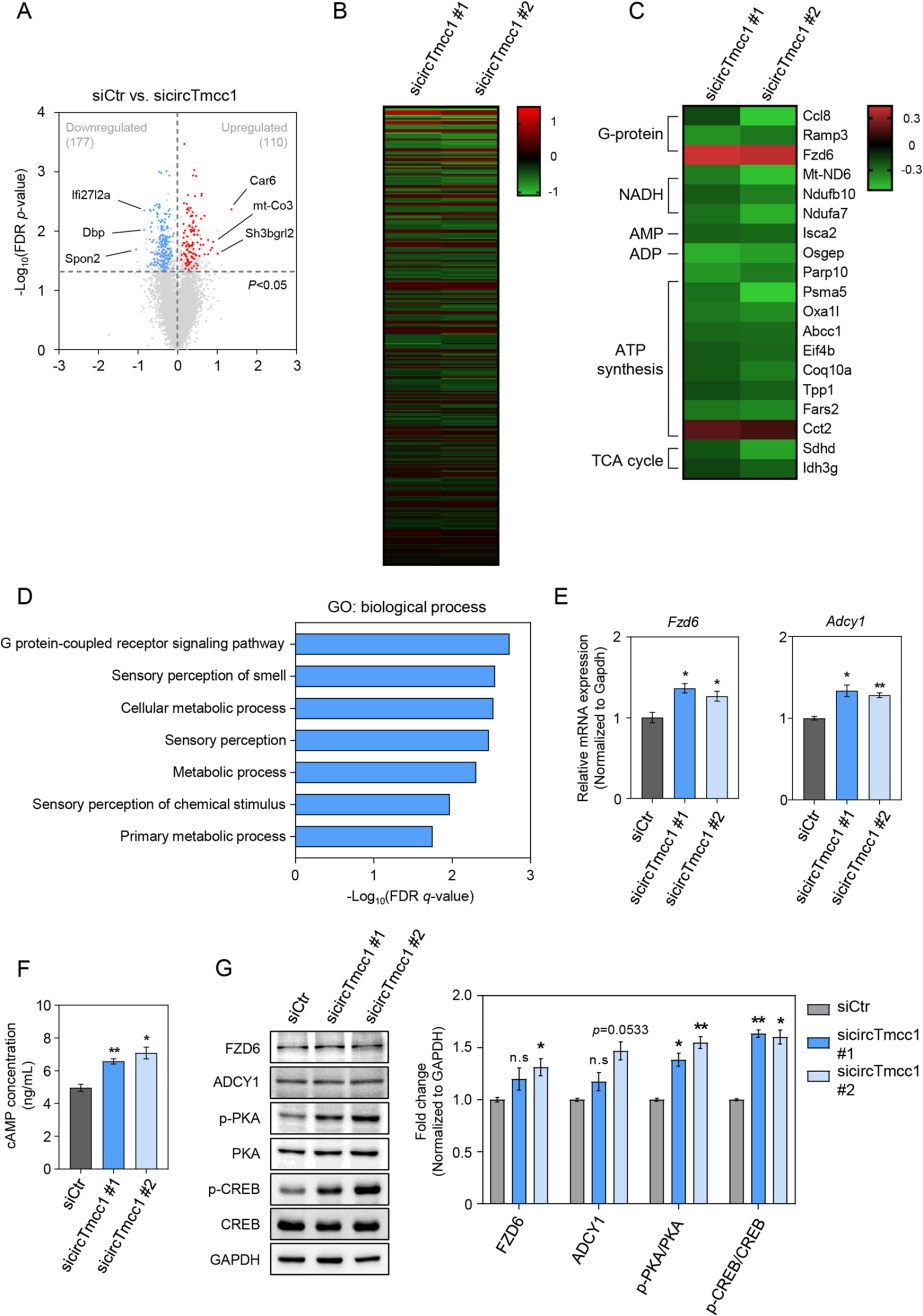
circTmcc1 regulates intracellular molecular mechanisms in hyperammonemia-exposed astrocytes. **A** Volcano plot for gene with altered expression changes that differed by knockdown of circTmcc1 in C8-D1a cells exposed to ammonia. Those genes with *p* < 0.05 and with > 50% expression changes are indicated by colored dots. siCtr indicates control siRNA whereas sicircTmcc1 indicates circTmcc1 siRNA. **B** Gene expression following the circTmcc1 knockdown in C8-D1a cells. **C** The gene expression changes related to GPCR and cellular metabolism following circTmcc1 knockdown. In **B** and **C**, the heat map represents the difference in the expression compared to that in the negative control siRNA-treated C8-D1a cells. **D** The Gene Ontology (GO) analysis of differentially expressed 287 protein-coding genes in volcano plot. The top 10 GO terms of biological process appear in FDR *q*-value. **E** The changes in cAMP-related mRNA (Fzd6 and Adcy1) levels following circTmcc1 knockdown in C8-D1a cells were measured and represented as the mean ± SEM (*n* = 3). **F** Measurement of cAMP concentration in C8-D1a cells after circTmcc1 knockdown. The data are expressed as three independent experiments in duplicate. **G** The measurement of FZD6, ADCY1, p-PKA and p-CREB protein levels following circTmcc1 knockdown in C8-D1a cells were measured and are depicted as the mean ± SEM (*n* = 3). p-PKA and p-CREB protein levels compared with total PKA and CREB protein levels. An unpaired two-tailed *t*-test with Welch’s correction was used for statistical analysis. ns, not significant, *p < 0.05, **p < 0.01

Based on the results of RNA-sequencing analysis, we predicted the molecular mechanisms of circTmcc1 in astrocytes. Transcriptomic analysis was performed using two independent algorithms to select genes whose expression was specifically changed by two types siRNAs of circTmcc1. We identified 110 upregulated genes and 177 downregulated genes following the inhibition of circTmcc1 (Fig. 3A). Based on RNA-seq data, among the genes whose expression was changed by the deletion of circTmcc1, we found that specific signaling pathways, such as G-protein-coupled receptor (GPCR) signaling, and cellular metabolism showed significant changes (Fig. 3B, C). Considering biological processes in protein-coding gene analysis using GO analysis, we assume that circTmcc1 is related to multiple metabolic processes in astrocytes (Fig. 3D). GPCR-based signaling is associated with cAMP production [46] and has been reported to play critical roles in the regulation of neurotransmitter secretion in astrocyte homeostasis and neuronal networks [47]. We measured mRNA levels of GPCRs Frizzled class receptor 6 (Fzd6) and *Adcy1*, one of the adenylyl cyclase (ADCY) families that catalyzes cAMP production from adenosine triphosphate (ATP). The expression of *Fzd6* and *Adcy1* mRNAs increased upon inhibition of circTmcc1 (Fig. 3E), which are known to be involved in cell growth, migration, and cellular metabolism through cAMP/PKA signaling [48]. To confirm that circTmcc1 regulates the concentration of intracellular cAMP, we measured cAMP concentration in astrocytes using a cAMP ELISA kit (Fig. 3F). The concentration of intracellular cAMP in astrocyte increased according to the suppression of circTmcc1 (Fig. 3F). Moreover, cAMP signaling in astrocytes is related to the protection of neuronal cells and the functional formation of neuronal networks [49]. It was confirmed that the protein levels of FZD6 and ADCY1 were increased by circTmcc1 inhibition. CircTmcc1 depletion significantly increased the expression of the phosphorylated protein kinase A (PKA) and cAMP response element-binding protein (CREB) in the C8-D1a astrocyte under hyperammonemic conditions (Fig. 3G). Taken together, circTmc1 is thought to modulate astrocytic function by regulating cAMP synthesis from ATP through the GPCR signaling pathway.

### circTmcc1 contributes to EAAT2 expression by modulating the p65 NF-κB transcriptional repressor complex

**Fig. 4:**
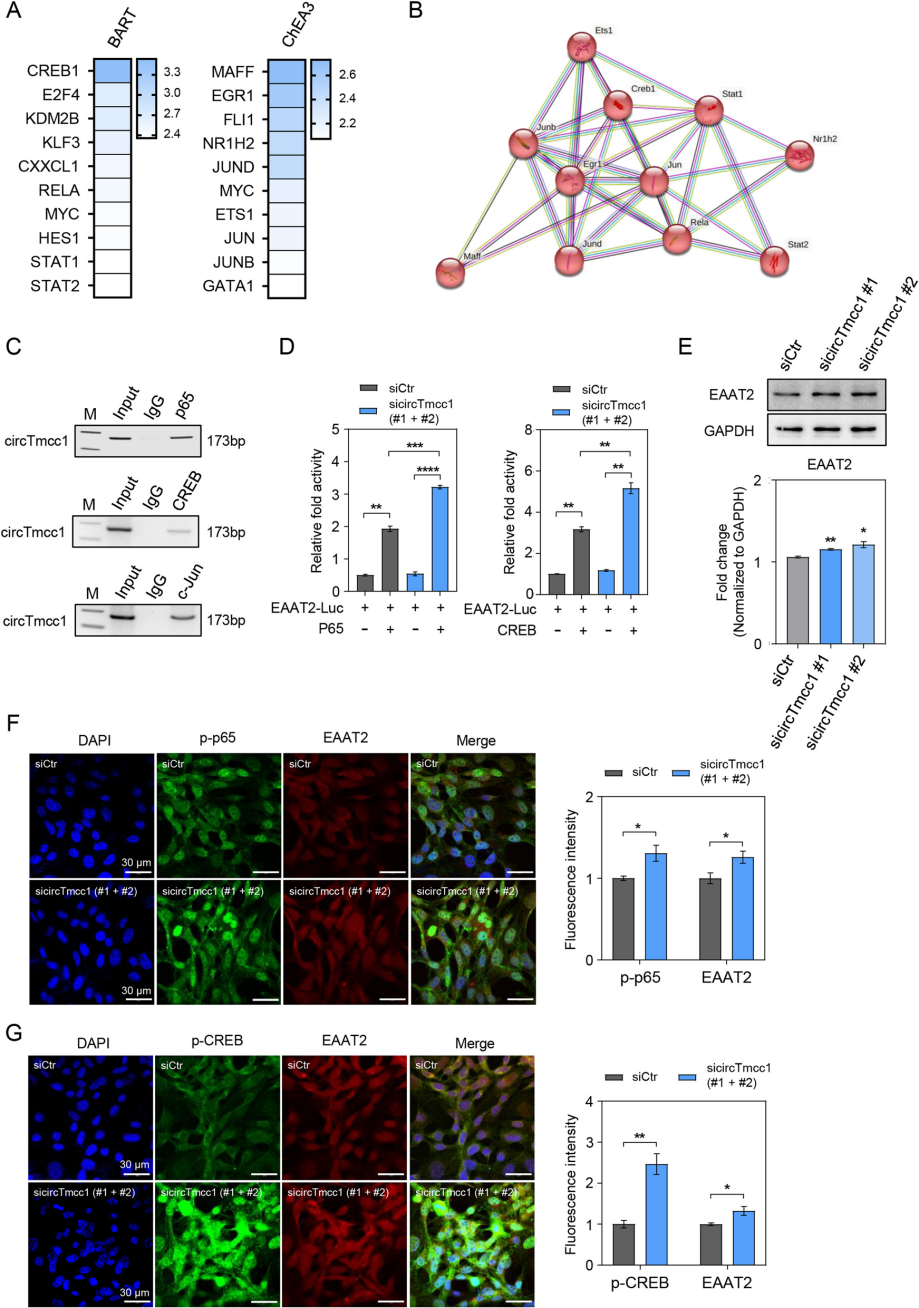
circTmcc1 regulates EAAT2 transcriptions via the p65-NF-κB transcriptional activator complex. **A** Prediction of a protein factor that can regulate the transcription of the gene affected by circTmcc1 downregulation. The color bars exhibited the BART evaluation using the Irwin–Hall *p*-value and the ChEA3 evaluation using the integrated Score Rank, respectively. **B** The possibility of interaction between circTmcc1 and the transcription factor was predicted by STRING. **C** The interaction between circTmcc1 and p65 NF-κB, CREB, and c-Jun were confirmed through RNA–protein interaction in C8-D1a cells. **D** 293T cells were transfected with the mouse EAAT2 (*Slc1a2*) promoter luciferase construct, along with expression vectors for p65 and CREB following circTmcc1 knockdown. The changes of EAAT2 (Slc1a2) promoter activity were measured and reported as the mean ± SEM (*n* = 3). **E** Confirmation of changes in the expression of EAAT2 protein, a glutamate transporter in C8-D1a cells treated with circTmcc1 siRNAs, are described as the mean ± SEM (*n* = 3). **F** Immunocytochemical images of p-NF-κB/EAAT2 expression in circTmcc1-downregulated C8-D1a cells. The representative cells from three independent cultures (*n* = 3). **G** Immunocytochemical images of CREB/EAAT2 expression in circTmcc1-downregulated C8-D1a cells. The representative cells from three independent cultures (*n* = 3). An unpaired two-tailed *t*-test with Welch’s correction was used for statistical analysis. ns, not significant, *p < 0.05, **p < 0.01, ***p < 0.001, ****p < 0.0001

To verify the mechanism related to transcriptional regulation of circTmcc1, we identified transcriptional factors or chromatin regulators that regulate differentially expressed genes in RNA-seq data. Two publicly available prediction web tools—BART and ChEA3—were used to analyze transcriptional factors and chromatin regulators containing transcriptional regulation of 287 genes related to the metabolic process (Fig. 3A–C). The top 10 transcription factors (Fig. 4A) predicted by both tools, BART (http://bartweb.ort) and ChEA3 (http://maayanlab.cloud/chea3/), were applied to the RNA-binding protein prediction RPIseq tool to predict the transcription factors that are likely to interact with circTmcc1 (Additional file 1: Fig. S8). The publicly available STRING web tool (http://string-db.org/) was used to confirm the interaction between circTmcc1 and the associated binding factors (Fig. 4B). The genes that interact with CREB that are regulated by circTmcc1 were identified, and binding with circTmcc1 was verified through RNA-IP assay. We confirmed that circTmcc1 binds to RELA (nuclear factor kappa-light-chain-enhancer of activated B cells, p65 NF-κB), CREB, and transcription factor jun (c-Jun; Fig. 4C and Additional file 1: Fig. S9A–C). Interestingly, p65 plays important roles in the regulation of EAAT2 expression [50], as an astrocyte glutamate transport marker, and connects with the binding promoter of EAAT2 [51]. Similar to p65, CREB regulates the expression of EAAT2 by binding to the promoter of EAAT2 [52]. Luciferase assay was performed to identify whether the depletion of circTmcc1 adjusts the binding to the promoter of EAAT2 by p65 and CREB. The overexpression of p65 and CREB enhanced EAAT2 promoter activity (Fig. 4D). Moreover, the depletion of circTmcc1 by sicircTmcc1 co-transfection increased p65 and CREB-induced EAAT2 promoter activity in 293T cells (Fig. 4D). We confirmed that the suppression of circTmcc1 leads to increased protein levels of the EAAT2 in astrocytes. CircTmcc1 knockdown induced increased expression of EAAT2 in C8-D1a astrocytes under hyperammonemic conditions (Fig. 4E). The fluorescence intensity of p-p65, p-CREB, and EAAT2 were higher in circTmcc1-depleted astrocytes than in the control siRNA-treated cells (Fig. 4F, G). These results indicate that circTmcc1 suppression increased the EAAT2 expression through binding of the EAAT2 promoter of p65 and CREB. Taken together, circTmcc1 deletion regulates p65- and CREB-mediated activation of EAAT2.

### circTmcc1 downregulates the secretion of proinflammatory cytokines and chemokines in astrocytes

**Fig. 5:**
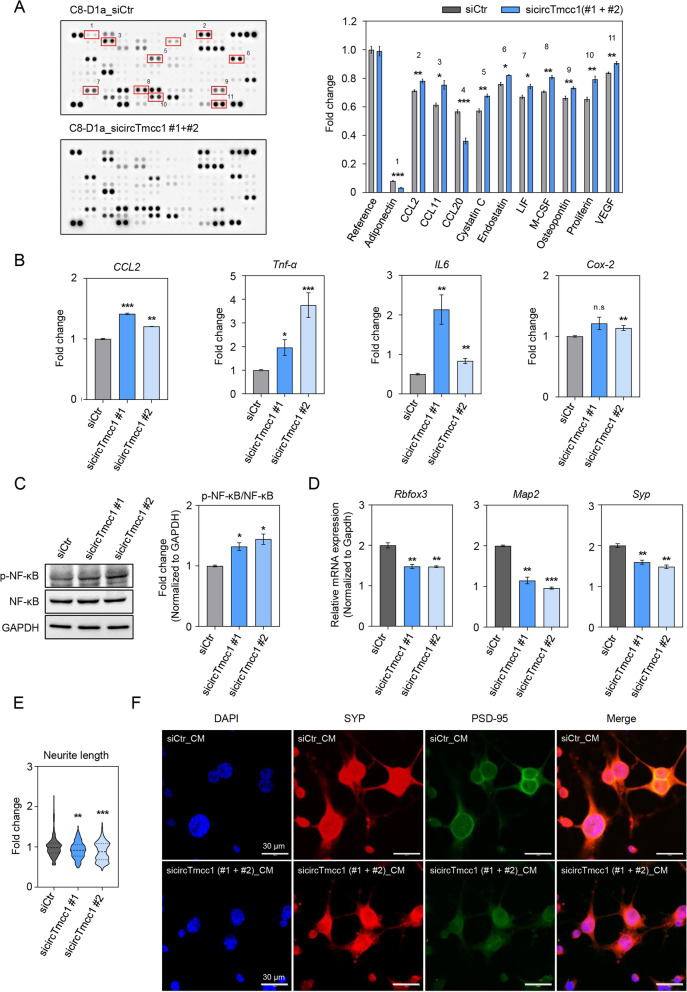
circTmcc1 controls the secretion of proinflammatory mediators in astrocytes and modulates neuronal complexity. **A** Immunoblot measurement of the cytokine and chemokine levels of control siRNA and circTmcc1-siRNA-transfected C8-D1a cells. Differential level histogram of cytokines and chemokines between control siRNA and circTmcc1-siRNA-treated cells. **B** The expression level of proinflammatory cytokine- and chemokine-related genes (Ccl2, Tnf-α, IL-6, IL-1β, and Cox2) in C8-D1a cells following circTmcc1 knockdown. These expression changes are described as the mean ± SEM (*n* = 3). **C** The changes in the protein of NF-κB following circTmcc1 knockdown in C8-D1a cells are described as the mean ± SEM (*n* = 3). **D** The gene expression changes (Rbfox3, Map2, and Syp) related to neuronal and synaptic function were measured and are presented as the mean ± SEM (*n* = 3). **E** Changes in the neurite length of the C8-D1a-conditioned media (CM)-treated differentiated Neuro-2A cells. **F** Immunocytochemical images of SYP and PSD-95 expression in circTmcc1-downregulated C8-D1a cells. The representative cells from three independent cultures (*n* = 3). An unpaired two-tailed *t*-test with Welch’s correction was used for statistical analysis. ns, not significant, *p < 0.05, **p < 0.01, ***p < 0.005

p65 is an important transcriptional factor in the inflammatory response [53], and p65 deletion by circTmcc1 affects the secretions of cytokines and chemokines in astrocytes [54]. We performed cytokine array analysis using cultured media (CM) supernatant in astrocytes treated with circTmcc1 siRNAs (#1 + #2) and control siRNA. We found that the circTmcc1 siRNAs-CM supernatant contained various proinflammatory cytokines and chemokines as compared to the control siRNA-CM (Fig. 5A). This result suggests that the inhibition of circTmcc1 promoted the release of proinflammatory cytokines and chemokines and further confirmed that circTmcc1 modulates the intracellular expression of inflammation-related genes. The expression of proinflammatory cytokines and chemokines, such as the C–C motif chemokine ligand 2 (*Ccl2*), tumor necrosis factor-α (*Tnf-α*), interleukin-6 (*IL-6*), and cyclooxygenase-2 (*Cox-2*) increased considerably in circTmcc1-depleted astrocytes (Fig. 5B). The phosphorylation of the p65 protein was upregulated in circTmcc1-depleted astrocytes under hyperammonemic conditions (Fig. 5C). These data indicate that circTmcc1 downregulates the intracellular and extracellular proinflammatory cytokine and chemokine secretions in hyperammonemia-exposed astrocytes.

### circTmcc1 improves neurite length and synaptic plasticity-related protein expression in neurons

We checked whether the factors secreted in astrocytes affects neuronal function and structure. We investigated neuronal function in neurons treated with CM-containing mediators that are secreted from astrocytes. mRNA levels of *Rbfox3*, *Map2*, and *Syp* were decreased by circTmcc1 siRNAs-CM-treatment in differentiated neurons as compared with differentiated neurons treated control siRNA-CM (Fig. 5D). To confirm the structural change of neurons, RA was treated to induce differentiation of Neuro-2A cells. We observed that the neurite length was reduced in the circTmcc1 siRNAs-CM-treated neurons compared to the control siRNA-CM-treated neurons (Fig. 5E). The fluorescence intensity of SYP and PSD-95 were higher in the control siRNA conditioned media-treated neurons than in the circTmcc1-depleted conditioned media-treated cells (Fig. 5F). These results suggested that cirTmcc1 regulates astrocytic secretion and subsequently influences neurite length and neuronal structures.

### circTmcc1 improves spatial memory in the BDL mouse brain

**Fig. 6:**
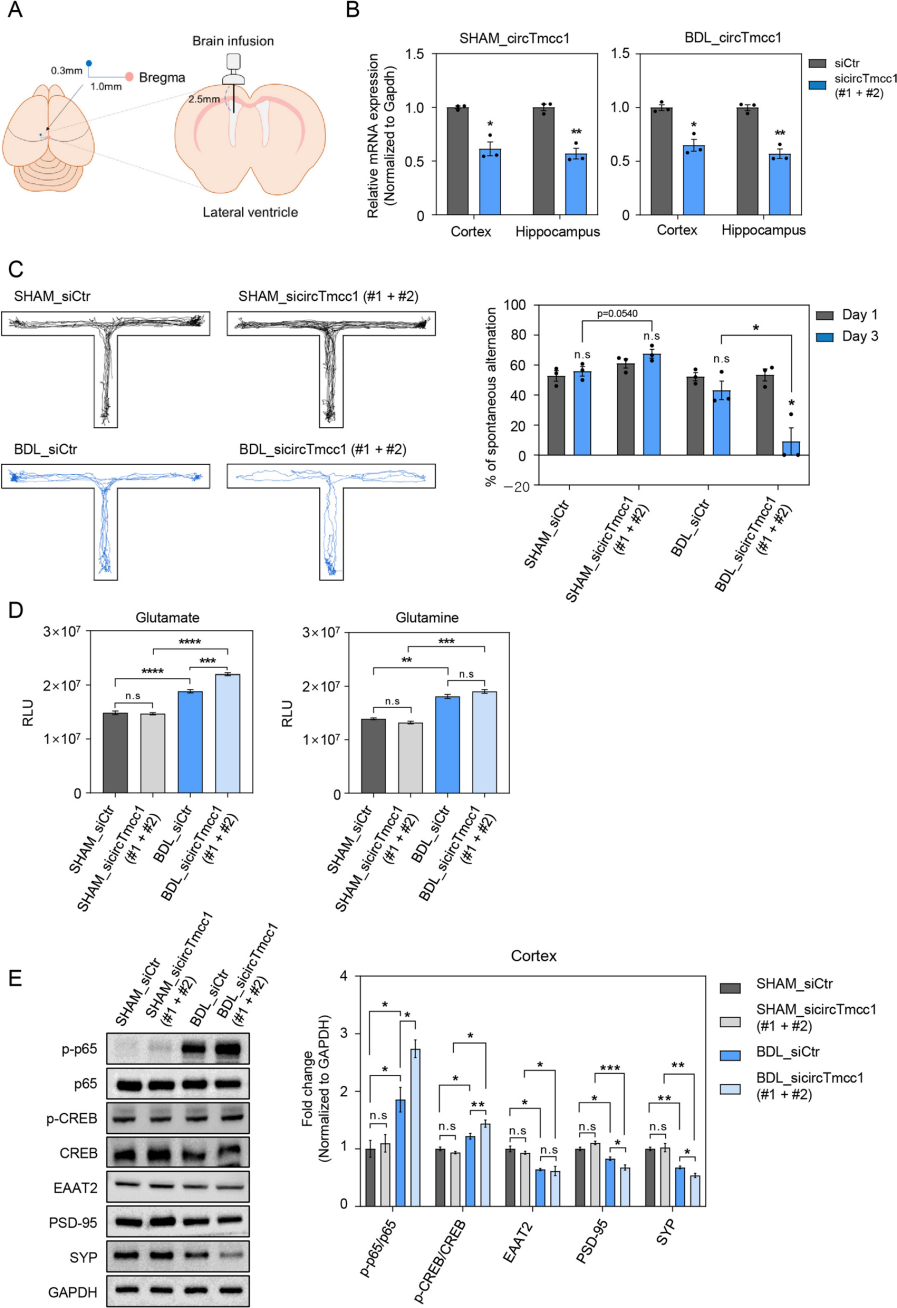
circTmcc1 regulates spatial memory in BDL mice. **A** Illustration of brain osmotic pump-infused mouse model. The osmotic pump was infused into the lateral ventricle (mediolateral 1.0 mm, anteroposterior 0.3 mm, and dorsoventral 2.5 mm, with the bregma as the landmark). **B** The expression of circTmcc1 and Tmcc1 in the cortex of sham and BDL mice, respectively, is presented as the mean ± SEM (*n* = 3). **C** An illustration describing the movement of sham and BDL mice in the T-maze apparatus following infusion from an osmotic pump filled with either control siRNA or circTmcc1-siRNA mixtures. **D** The measurement of glutamate and glutamine concentration in the sham and BDL mice cortex following circTmcc1 knockdown. **E** The expression of proteins in sham and BDL mice cortex following circTmcc1 knockdown. p-p65 and p-CREB protein levels compared with total p65 and CREB protein levels. Data are represented as the mean ± SEM (*n* = 3). An unpaired two-tailed *t*-test with Welch’s correction was used for statistical analysis. ns, not significant, *p < 0.05, **p < 0.01, ***p < 0.001, ****p < 0.0001

The regulation of cAMP signaling is a cardinal aspect of memory formation, synaptic plasticity, and glutamate transport in the brain [52, 55, 56]. Firstly, we measured the memory function of sham and BDL mice through behavioral tests after suppressing the expression of circTmcc1. For the suppression of circTmcc1 expression in mouse brain, two types of circTmcc1 siRNA mixtures were directly infused into the lateral ventricles of the brain (Fig. 6A). Using an osmotic pump, we infused siRNA into the brain 10 days after surgery. The levels of AST, ALT, total and direct bilirubin, and ammonia were increased in BDL mice than in sham mice (Additional file 1: Fig. S10A–C). However, siRNA pump infusion did not change these levels. Moreover, the preoperative body weight of the siRNA pump-infused sham and BDL mice was similar (28.3 ± 0.2 g in sham and BDL siRNA-infused all groups), whereas the postoperative body weight had significantly decreased in the BDL groups, regardless of siRNA pump infusion (30.7 ± 0.3 g vs. 29 ± 1 g vs. 21.3 ± 0.5 g vs. 20.6 ± 0.4 g in control siRNA, circTmcc1 siRNA-infused sham and BDL group, respectively; Additional file 1: Fig. S10D). We confirmed that circTmcc1 expression was reduced in the cortex and hippocampus of both sham and BDL mice after brain infusion of circTmcc1 siRNAs (Fig. 6B). To evaluate whether circTmcc1 affects the memory function in the mice, we conducted the T-maze behavior test to assess the spatial memory. Movement and locomotor activities were assessed using the T-maze to evaluate the spatial memory (Fig. 6C). However, circTmcc1 depletion did not influence the spatial memory in sham mice, and there was no significant change in memory capacity in sham mice that were infused with circTmcc1 siRNAs (Fig. 6C). In contrast, the spatial memory of circTmcc1 siRNA-infused BDL mice decreased compared to that of the control siRNA-infused BDL mice, and had significantly decreased on Day 3 compared to that on Day 1 in circTmcc1 siRNA-infused mice. This indicates that circTmcc1 depletion decreases spatial learning and memory function during trials in BDL mice. We found that the tendency of locomotor activities was slightly decreased in circTmcc1 siRNA-infused-BDL mice, which indicates that circTmc1 depletion affects movement and locomotor activities in BDL mice (Additional file 1: Fig. S11A–D).

To check whether glutamate metabolism in the brain is altered by circTmcc1 infusion, glutamate and glutamine levels in cortex lysate were measured (Fig. 6D). The glutamate level of the cortex of BDL mice was higher than that of SHAM mice, and also deletion of circTmcc1 in BDL mice leads to more increased level of glutamate. Glutamine concentration was higher in BDL mice compared to SHAM mice, but were not significantly changed by knockdown of circTmcc1 in BDL mice. These results indicate that circTmcc1 affects the accumulation of glutamate in the mouse cortex and influences the conversion system between glutamate and glutamine.

Furthermore, we measured the expression of phosphorylated p65, phosphorylated CREB, and EAAT2 in the cortex of SHAM and BDL mice after circTmcc1 depletion. We confirmed higher expression of phosphorylated p65 and CREB in the cortex of BDL mice than SHAM mice (Fig. 6E). Moreover, phosphorylated p65 and CREB were considerably increased by circTmcc1 knockdown in the cortex of BDL mice (Fig. 6E). The expression of EAAT2 protein was decreased in BDL mice than in SHAM mice regardless of circTmcc1 depletion (Fig. 6E). In addition, the expression of PSD-95 and SYP was significantly decreased in BDL mice compared to SHAM mice, and also the expression of PSD-95 and SYP was more decreased by circTmcc1 depletion (Fig. 6E). These results indicate that circTmcc1 influences spatial memory by modulating the synaptic plasticity-related proteins in hepatic encephalopathy brain.

## Discussion

In this study, we investigated whether circTmcc1, which is selected by RNA sequencing analysis in HE brain, contributes to astrocyte function and neuronal function in an in vitro hyperammonemic condition as well as in an in vivo BDL HE model. We found that the inhibition of circTmcc1 by siRNA increases the amount of glutamate and decreases the amount of glutamine by reducing glutamine synthetase in astrocytes, and increases the expression of the glutamate transporter enzyme *Gls* gene. Moreover, we confirmed that intracellular GABA concentration in astrocytes decreased by depletion of circTmcc1. These results indicate that circTmcc1 regulates the glutamine and GABA level in astrocytes and contributes to amino acids for metabolism of brain [57] under hyperammonemic conditions that are caused by HE. Furthermore, we observed increased expression of AQP4 as the astrocyte-swelling-related marker [45] involved in brain water balance by siRNA circTmcc1 treatment in astrocyte. Based on this data, we hypothesized that circTmcc1 could regulate the glutamate conversion capacity of astrocytes under HE-induced hyperammonemic conditions and thereby inhibit astrocytic swelling.

In the CNS, glutamate is involved in various neuronal functions, including the secretion of neurotransmitters and neuronal synapse plasticity [58]. Dysregulation of glutamate concentration in the extracellular space causes excessive excitability of postsynaptic neurons, and this is associated with neuropathological and neuropsychiatric diseases [59]. Additionally, excessive accumulation of glutamate in neuronal cells causes neuronal cell death and neuronal dysfunction [60], and ultimately leads to the onset of various neurodegenerative diseases, including Alzheimer’s disease (AD) [61]. Moreover, glutamine synthesized in astrocytes is released and taken up by neurons, and serves as a precursor of GABA through glutamate [62]. The transport of neurotransmitter in neurons and glutamine in astrocyte is called the glutamate/GABA–glutamine cycle, and its balance is essential for regulating brain metabolism and function [57]. Low concentrations of glutamine synthetase and glutamine have been confirmed in AD brains, and increased glutamine concentrations have been reported to protect neurons from amyloid beta (Aβ) induced neurotoxicity [63]. Furthermore, glutamate/GABA–glutamine cycle is involved in the regulation of energy metabolism and neurotransmitter homeostasis in astrocytes [64], and disruption of astrocyte metabolic function contributes to synaptic impairment [65]. Our data showed that circTmcc1 regulates astrocyte glutamate metabolism, and thus suggests that circTmcc1 may influence the onset and progression of neurological diseases.

Moreover, given our RNA sequencing data, inhibition of circTmcc1 changes the expression of genes that are related to intracellular metabolism. In particular, we confirmed the changes of gene expression related to GPCR and ATP synthesis due to inhibition of circTmcc1 in astrocytes. In our data, we found that the mRNA expressions of *Fzd6*, related to GPCR synthesis, and *Acyl1*, related to cAMP synthesis. were increased by inhibition of circTmcc1. We observed that the increased phosphorylation of PKA/CREB protein involved in cAMP signaling [66, 67] in hyperammonia subsequently induced astrocytes through inhibition of circTmcc1. The level of cAMP in the astrocyte and neuron interaction is very important, in that it changes synaptic plasticity and memory function by regulating the release of gliotransmitters [56]. Based on these data, we infer that circTmc1 modulates cellular metabolism and ATP synthesis in astrocytes through the cAMP/PKA/CREB signaling pathway. Considering our consequences, circTmcc1 could improve astrocyte glutamate homeostasis as well as energy metabolism and ATP synthesis signaling in astrocytes, which are involved in neuronal function such as memory function [68–71].

From our data, it is indicated that circTmcc1 regulates the p65 (NF-κB)-c-Jun-CREB transcriptional complex, and subsequently contributes to the deactivation of glutamate transporter EAAT2 by binding with the EAAT2 promoter. Although our RNP-IP experiments do not confirm the direct binding of circTmcc1 to proteins, it does show that they are part of a complex. Which proteins circTmcc1 directly binds to, and which sequences within circTmcc1 are important for protein binding, remain to be determined by future experiments such as electrophoretic mobility shift assay.p65 as a transcription factor has been known to play critical roles in immunity, inflammatory responses, cell proliferation, and cellular differentiation [53]. Furthermore, the alteration of EAAT2 expression indicates changes in astrocyte glutamate transporter expression, and altered glutamate-to-glutamine conversion ability [72–74]. One study mentioned that excessive glutamate that is accumulated by EAAT2 activity is converted to glutamine by glutamine synthetase (GS) and subsequently transported to neurons [75]. We assumed that the reduced EAAT2 expression mediated by circTmcc1 affects the homeostasis of glutamate metabolism in astrocytes, which are involved in memory function.

Collectively, we assume that circTmcc1 contributes to the improvement of cognitive decline by enhancing the imbalance of the glutamate/glutamine ratio in astrocytes, considering previous evidence of the relationship between glutamate ratio and memory function [76]. Moreover, the inhibition of circTmcc1 promotes the secretion of proinflammatory mediators, such as TNF-α and IL-6, as evidenced in our data. In addition, the supernatant obtained from siRNA circRNA Tmcc1-treated astrocytes causes neural complexity and synaptic dysfunction in neurons. Thus, we assume that circTmcc1 inhibition leads to neuronal synaptic dysfunction by promoting the secretion of proinflammatory mediators in hyperammonia-induced astrocytes. The regulation of inflammatory mediator secretion in astrocytes is linked to the neural synaptic formation and altered patterns of neurotransmitter secretion [77]. Our data revealed that circTmcc1 may regulate cognitive function in hyperammonemic environments by modulating the secretory pattern of inflammatory mediators in astrocytes.

These data suggest that circTmcc1 could modulate the cellular inflammatory response in astrocytes and simultaneously control neuronal function. We speculate that circTmcc1 may regulate the interactive signaling between neurons and glia. Furthermore, based on the results of our T-maze behavior test and Western blotting data, we assume that circTmcc1 improves spatial memory ability by enhancing the expression of synaptic function-related markers, such as c-Fos, PSD-95, and SYP, both in the in vitro neuronal cells and in the in vivo BDL mouse brain, thereby suggesting that synaptic plasticity and neuronal function affect memory formation and cognitive function [78, 79]. This increased CREB and p65 interaction synergistically recruits CBP and leads to greater activation of the transcription of multiple genes [80].

Furthermore, p65 binds with other members of the CREB family, including ATF-2 and c-jun [81], and the CREB promoter comprises binding sites for CREB adjacent to the p65 motif [82]. Therefore, as one of the components of p65, the activation of CREB is critical for the nuclear translocation of p65 [83]. The p65 and CREB complex regulates the expression of glutamate transporters in astrocytes [52], and subsequently influences the transcription of neuronal cell damage and synaptic function-related genes [84, 85], which leads to changes in the learning and memory function [86, 87]. A previous study reported that increased p65 translocation into the nucleus is linked to the neuroinflammation [88] and memory impairment [89]. Based on our data, we infer that circTmcc1 could improve spatial memory ability through p65 and CREB signaling in the HE brain. Also, although the protein level of EAAT2 in brain tissue did not change significantly between the two groups, these data suggest that the brain tissue contains many types of cells as well as astrocytes. Taken together, we suggest that circTmcc1 influences astrocytic glutamate metabolism and spatial memory ability by controlling NF-κB p65 and CREB signaling.

In conclusion, we summarize that circTmcc1 regulates cellular and glutamate metabolism homeostasis and modulates the inflammatory response in astrocytes through p65 CREB signaling activation under HE-induced hyperammonia conditions. Finally, the circTmcc1-mediated regulation in astrocyte function improves spatial memory ability and synaptic plasticity in the HE brain. Thus, we suggest that modulation of circTmcc1 in the brain may be a novel therapeutic approach for solving neuropathological issues in the HE brain.

## Supplementary Information

**Additional** **file** **1:** **Figure** **S1.** Liver function-related markers changes in BDL mice plasma. Measurement of AST and ALT level in both sham and BDL mice plasma. Measurement of Total bilirubin and Direct bilirubin level in both sham and BDL mice plasma. Quantification of ammonia level in the sham and BDL mice plasma. In **a**–**c,** data are presented as mean ± SEM. Measurement of body weight of sham and BDL mice after bile duct ligation surgery. An unpaired two-tailed *t*-test with Welch’s correction was used for statistical analysis. Ns, not significant, *p < 0.05, **p < 0.01. **Figure** **S2.** Selection of differentially expressed circular RNAs in the brain cortices of BDL mice. The expression level of each circRNA between sham and BDL mice. The fold-change in the circRNA expression was normalized to circRNA expression counts of sham mice. **Figure** **S3.** Cell type-specific expression of hepatic encephalopathy-related circTmcc1 release in the brain. The specific expression of circTmcc1 in Neuro-2A, BV2, and C8-D1a. The measurement of circTmcc1 expression was performed and reported as the mean ± SEM with a relative percentage in each cell. Confirmation of the circular structure of circTmcc1 in Neuro-2A andBV2 cells. **Figure** **S4.** The human Tmcc1 expression in Genotype Tissue Expression. Data are described as the expression level of the median gene in 52 tissues and 2 cell lines of human origin based on RNA-sequencing data from the GTEx final data release. The data are presented as transcripts per million. **Figure** **S5.** Tissue type-specific expression of circTmcc1 and Tmcc1 in the brain. The expression of circTmcc1 and Tmcc1 in the brain cortex, hippocampus, Striatum of sham and BDL mice. The data are presented as the mean ± SEM. An unpaired two-tailed *t*-test with Welch’s correction was used for statistical analysis. The expression of circTmcc1 and Tmcc1 in the lung, liver, muscle, fat, spleen, gut, kidney of mice. The data are presented as the mean ± SEM. ns, not significant, *p < 0.05. **Figure** **S6.** Subcellular localization of circTmcc1 and knockdown efficiency of circTmcc1 in the brain. The distribution of circTmcc1 and Tmcc1 in the nucleus and cytoplasm of Neuro-2A, BV2, and C8-D1a cells, respectively. The expression measured from three independent experiments and the relative difference between the nuclear and cytoplasmic expressions is shown as a percentage ratio. Pre-GAPDH and GAPDH were used for controls of nuclear RNA and cytoplasmic RNA, respectively. The expression of circTmcc1 and Tmcc1 in each cell type under hyperammonemic conditions are reported as the mean ± SEM. Changes in the expression of circTmcc1 and Tmcc1 following cirTmcc1 knockdown in each cell type are presented as the mean ± SEM. siCtr indicates the control of siRNA treatment. An unpaired two-tailed *t*-test with Welch’s correction was used for statistical analysis. ns, not significant, *p < 0.05, **p < 0.01, ***p < 0.005. **Figure** **S7.** Functional changes of neuron and microglia by circTmcc1 knockdown. Changes in morphology and neurite complexity following circTmcc1 knockdown in Neuro-2A cells and data are described as Mean ± SEM. The expression of neuronal function-related genes following circTmcc1 knockdown in Neuro-2A cells is presented as the ±SEM. The expression of inflammation-related genes following circTmcc1 knockdown in BV2 cells is presented as ±SEM. An unpaired two-tailed *t*-test with Welch’s correction was used for statistical analysis., Immunocytochemical images of AQP4 expression in circTmcc1 downregulated C8-D1a cells. The representative cells from three independent cultures. ns, not significant, *p < 0.05, **p < 0.01, ***p < 0.001. **Figure** **S8.** The probability of interaction between circTmcc1 and specific transcriptional factor. RPIseq was used to confirm the interaction between circTmcc1 and the transcription regulator. RF and SVM refer to random forest and support vector forest, respectively. The color bars show the possibility score of interaction. **Figure** **S9.** RNA-binding protein immunoprecipitation of circTmcc1.Interaction between circTmcc1 and p65 NF-κB.Interaction between circTmcc1 and CREB. Interaction between circTmcc1 and c-Jun. M indicates the RNA size ladder. The expression of p65 NF-κB protein in cell lysates after immunoprecipitation with magnetic beads and antibody complexes. **Figure** **S10.** Liver function-related markers changes in BDL mice plasma. SHAM-siCtr indicates control siRNA-infused sham mice, and sham-sicircTmcc1indicates the sham mice infused with two circTmcc1 siRNAs. BDL_siCtr indicates control siRNA-infused BDL mice, whereas BDL_sicircTmcc1indicates the BDL mice infused with two circTmcc1 siRNAs. Data are described as the mean ± SEM. Measurement of ALT and AST levels in the plasma of sham and BDL mice following siRNA pump infusion. Measurement of total and direct bilirubin levels in the plasma of both sham and BDL mice following siRNA pump infusion. Quantification of ammonia level in the sham and BDL mice plasma following siRNA pump infusion. Measurement of body weight of siRNA osmotic pump-infused sham and BDL mice after bile duct ligation surgery. **Figure** **S11.** The locomotor activities of brain osmotic pump-infused sham and BDL mice. SHAM_siCtr indicates control siRNA-infused sham mice, and sham-sicircTmcc1indicates the sham mice infused with two circTmcc1 siRNAs. BDL_siCtr indicates control siRNA-infused BDL mice, and BDL_sicircTmcc1indicates the BDL mice infused with two circTmcc1 siRNAs. The changes in the number of entrances to the arms, the changes in the total length of mouse movement, the changes in the immobility time of mouse, and the changes in the velocity of mouse movement, for sham and BDL mice following pump infusion of either control siRNA or circTmcc1 siRNAs, respectively. Data were measured at days 1 and 3 during the experiment and are reported as the mean ± SEM.

**Additional** **file** **2:** **Table** **S1.** Primer list.

**Additional** **file** **3:** **Table** **S2.** Expression level of circRNAs differentially expressed in BDL mouse cortex.

**Additional** **file** **4:** **Table** **S3.** BART, ChEA3 top 10 gene.

## Data Availability

The datasets used and/or analyzed during the current study are available from the corresponding author upon reasonable request.

## Abbreviations

circRNA: Circular RNAs
ncRNAs: Non-coding RNA
BDL: Bile duct ligation
HE: Hepatic encephalopathy
CNS: Central nervous system
BBB: Blood–brain barrier
GO: Gene Ontology
ALT: Alanine transaminase activity
AST: Aspartate amino aminotransferase
NH_4_Cl: Ammonium chloride
siRNAs: Small-interfering RNAs
AQP4: Aquaporin-4
GPCR: G-protein-coupled receptor
ATP: Adenosine triphosphate
PKA: Phosphorylated protein kinase A
CREB: CAMP response element-binding protein
NF-κB: Nuclear factor kappa-light-chain-enhancer of activated B cells
AD: Alzheimer’s disease
