# Supplementary material for: Circular RNA Tmcc1 improves astrocytic glutamate metabolism and spatial memory via NF-κB and CREB signaling in a bile duct ligation mouse model: transcriptional and cellular analyses
Source: J Neuroinflammation. 2023 May 22;20:121. doi: 10.1186/s12974-023-02806-w (PMC10204305; doi:10.1186/s12974-023-02806-w)
Supplement: Supplementary file 1 — Additional file 1: Figure S1. Liver function-related markers changes in BDL mice plasma. Measurement of AST and ALT level in both sham and BDL mice plasma. Measurement of Total bilirubin and Direct bilirubin level in both sham and BDL mice plasma. Quantification of ammonia level in the sham and BDL mice plasma. In a–c, data are presented as mean ± SEM. Measurement of body weight of sham and BDL mice after bile duct ligation surgery. An unpaired two-tailed t-test with Welch’s correction was used for statistical analysis. Ns, not significant, *p < 0.05, **p < 0.01. Figure S2. Selection of differentially expressed circular RNAs in the brain cortices of BDL mice. The expression level of each circRNA between sham and BDL mice. The fold-change in the circRNA expression was normalized to circRNA expression counts of sham mice. Figure S3. Cell type-specific expression of hepatic encephalopathy-related circTmcc1 release in the brain. The specific expression of circTmcc1 in Neuro-2A, BV2, and C8-D1a. The measurement of circTmcc1 expression was performed and reported as the mean ± SEM with a relative percentage in each cell. Confirmation of the circular structure of circTmcc1 in Neuro-2A andBV2 cells. Figure S4. The human Tmcc1 expression in Genotype Tissue Expression. Data are described as the expression level of the median gene in 52 tissues and 2 cell lines of human origin based on RNA-sequencing data from the GTEx final data release. The data are presented as transcripts per million. Figure S5. Tissue type-specific expression of circTmcc1 and Tmcc1 in the brain. The expression of circTmcc1 and Tmcc1 in the brain cortex, hippocampus, Striatum of sham and BDL mice. The data are presented as the mean ± SEM. An unpaired two-tailed t-test with Welch’s correction was used for statistical analysis. The expression of circTmcc1 and Tmcc1 in the lung, liver, muscle, fat, spleen, gut, kidney of mice. The data are presented as the mean ± SEM. ns, not significant, *p < 0.05. Figure S6. [file 12974_2023_2806_MOESM1_ESM.docx]

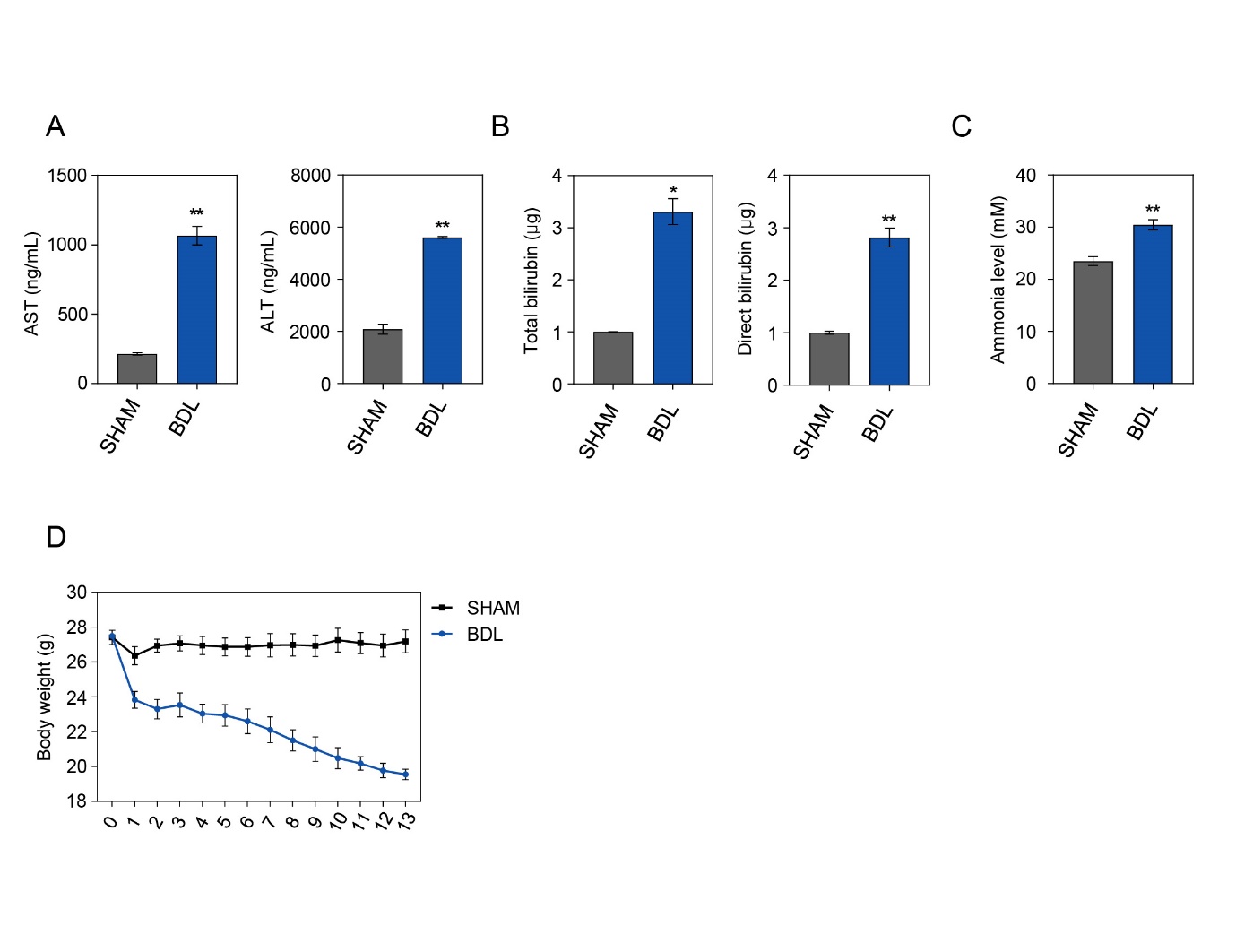


**Figure S1. Liver function-related markers changes in BDL mice plasma. (a)** Measurement of AST and ALT level in both sham and BDL mice plasma. (**b)** Measurement of Total bilirubin and Direct bilirubin level in both sham and BDL mice plasma. (**c)** Quantification of ammonia level in the sham and BDL mice plasma. In **a–c,** data are presented as mean ± SEM (*n* = 5). (**d**) Measurement of body weight of sham and BDL mice after bile duct ligation surgery. An unpaired two-tailed *t*-test with Welch’s correction was used for statistical analysis. Ns, not significant, **p* < 0.05, ***p* < 0.01.


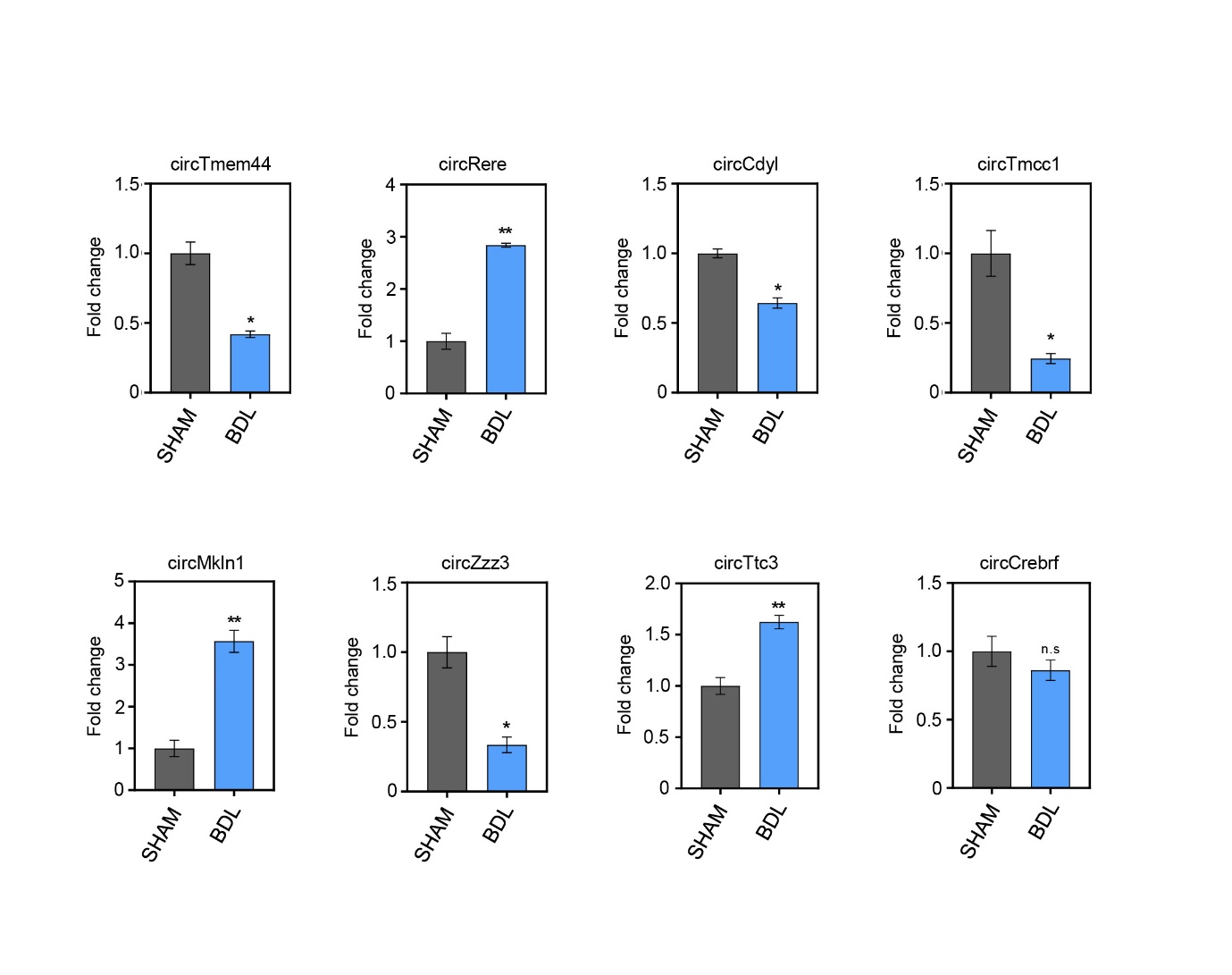


**Figure S2. Selection of differentially expressed circular RNAs in the brain cortices of BDL mice.** The expression level of each circRNA between sham and BDL mice. The fold-change in the circRNA expression was normalized to circRNA expression counts of sham mice.


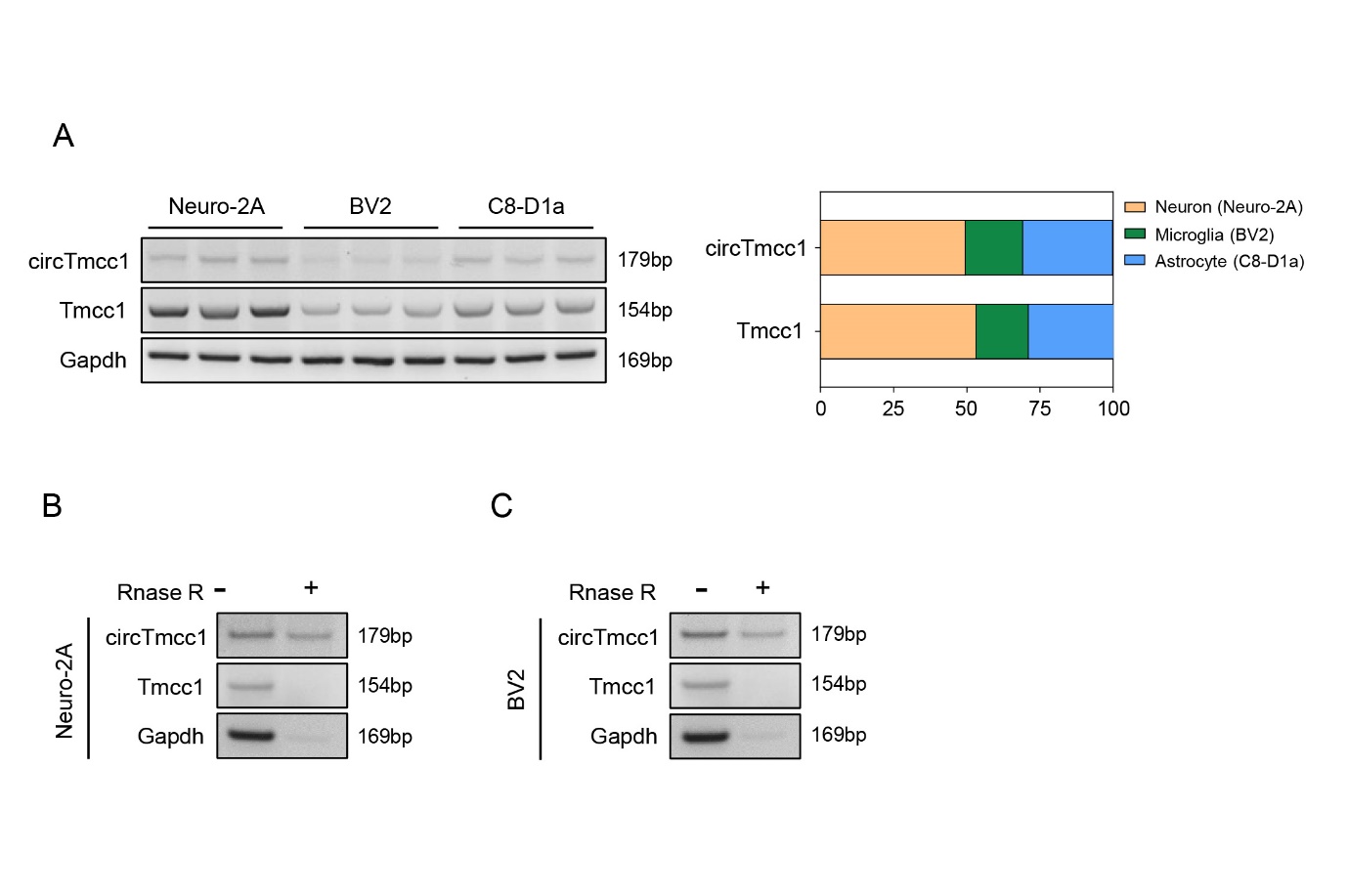


**Figure S3. Cell type-specific expression of hepatic encephalopathy-related circTmcc1 release in the brain.** (**a**) The specific expression of circTmcc1 in Neuro-2A, BV2, and C8-D1a. The measurement of circTmcc1 expression was performed and reported as the mean ± SEM (*n* = 3) with a relative percentage in each cell. (**b**) Confirmation of the circular structure of circTmcc1 in Neuro-2A and (**C**) BV2 cells.


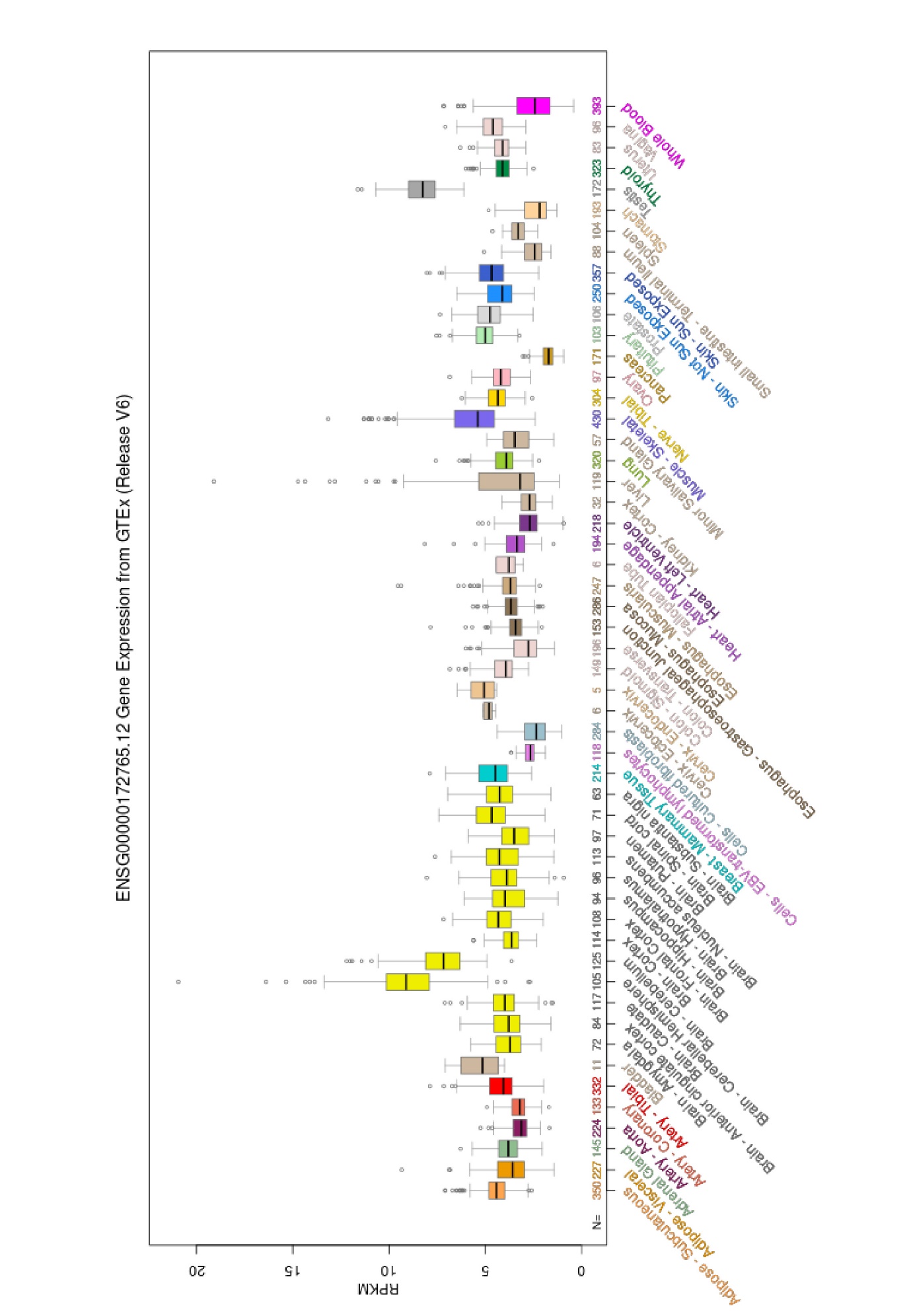


**Figure S4. The human Tmcc1 expression in Genotype Tissue Expression (GTEx).** Data are described as the expression level of the median gene in 52 tissues and 2 cell lines of human origin based on RNA-sequencing data from the GTEx final data release (V8). The data are presented as transcripts per million (TPM).


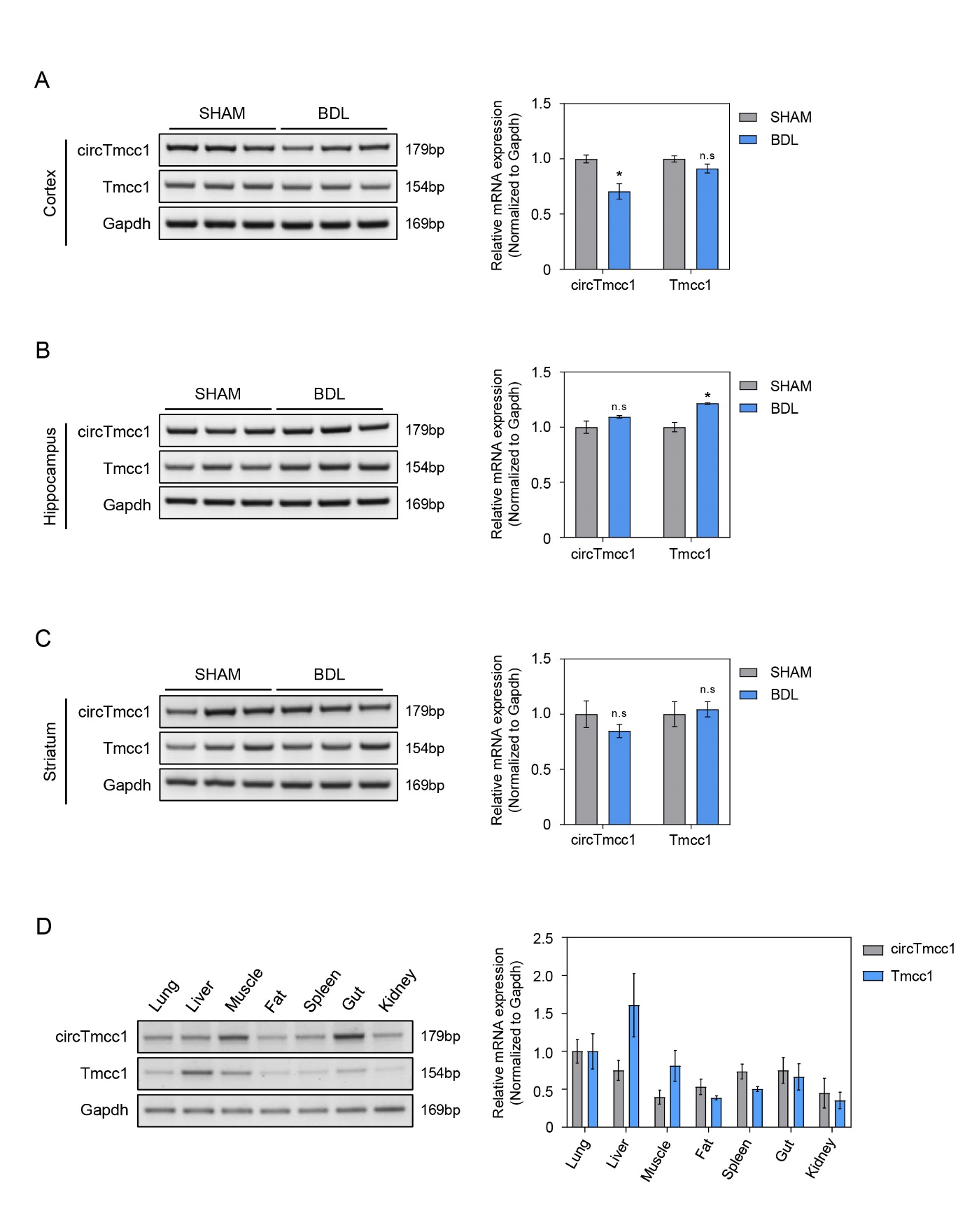


**Figure S5. Tissue type-specific expression of circTmcc1 and Tmcc1 in the brain.** The expression of circTmcc1 and Tmcc1 in the brain (**a**) cortex, (**b**) hippocampus, (**c**) Striatum of sham and BDL mice. The data are presented as the mean ± SEM (*n* = 3). An unpaired two-tailed *t*-test with Welch’s correction was used for statistical analysis. (**d**) The expression of circTmcc1 and Tmcc1 in the lung, liver, muscle, fat, spleen, gut, kidney of mice. The data are presented as the mean ± SEM (*n* = 3). ns, not significant, **p* < 0.05.


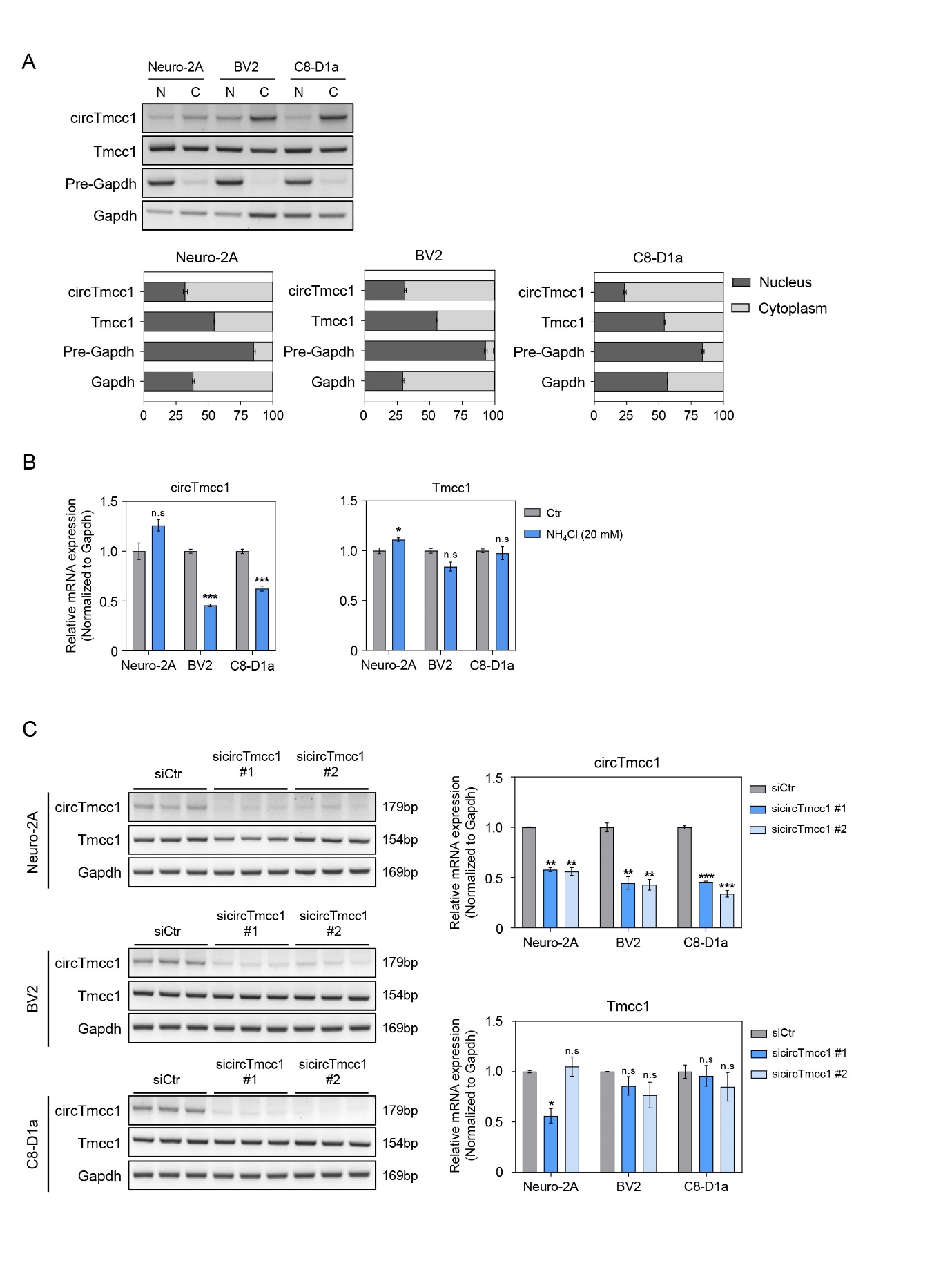


**Figure S6. Subcellular localization of circTmcc1 and knockdown efficiency of circTmcc1 in the brain.** (**a**) The distribution of circTmcc1 and Tmcc1 in the nucleus (N) and cytoplasm (C) of Neuro-2A, BV2, and C8-D1a cells, respectively. The expression measured from three independent experiments and the relative difference between the nuclear and cytoplasmic expressions is shown as a percentage ratio. Pre-GAPDH and GAPDH were used for controls of nuclear RNA and cytoplasmic RNA, respectively. (**b**) The expression of circTmcc1 and Tmcc1 in each cell type under hyperammonemic conditions are reported as the mean ± SEM (*n* = 3). (**c**) Changes in the expression of circTmcc1 and Tmcc1 following cirTmcc1 knockdown in each cell type are presented as the mean ± SEM (*n* = 3). siCtr indicates the control of siRNA treatment. An unpaired two-tailed *t*-test with Welch’s correction was used for statistical analysis. ns, not significant, **p* < 0.05, ***p* < 0.01, ****p* < 0.005.


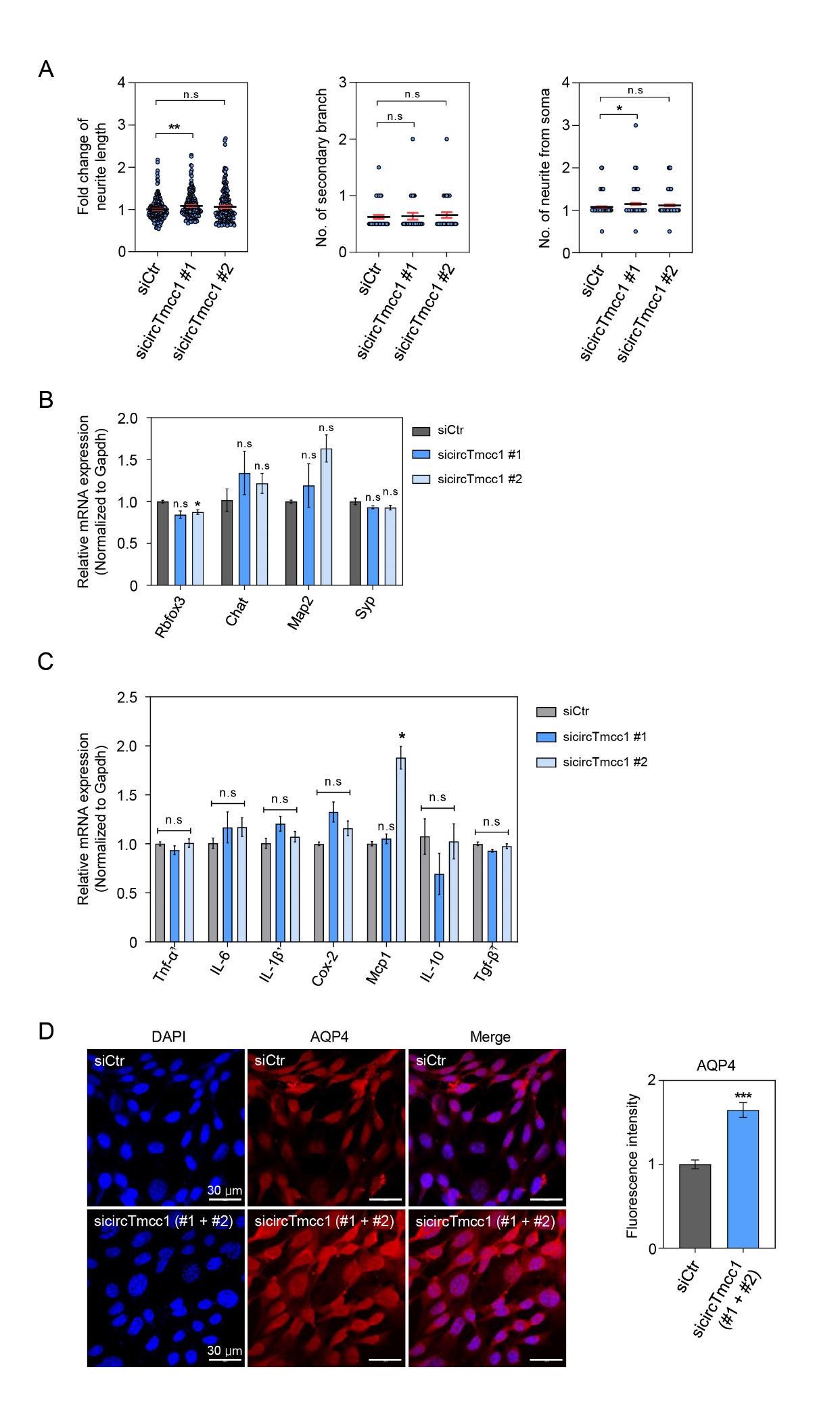


**Figure S7. Functional changes of neuron and microglia by circTmcc1 knockdown.** (**a**) Changes in morphology and neurite complexity following circTmcc1 knockdown in Neuro-2A cells and data are described as Mean ± SEM (*n* = 3). (**b**) The expression of neuronal function-related genes (Rbfox3, Chat, Map2, and Syp) following circTmcc1 knockdown in Neuro-2A cells is presented as the ±SEM (*n* = 3). (**c**) The expression of inflammation-related genes following circTmcc1 knockdown in BV2 cells is presented as ±SEM (*n* = 3). An unpaired two-tailed *t*-test with Welch’s correction was used for statistical analysis. (**d**), Immunocytochemical images of AQP4 expression in circTmcc1 downregulated C8-D1a cells. The representative cells from three independent cultures (*n* = 3). ns, not significant, **p* < 0.05, ***p* < 0.01, ****p* < 0.001.


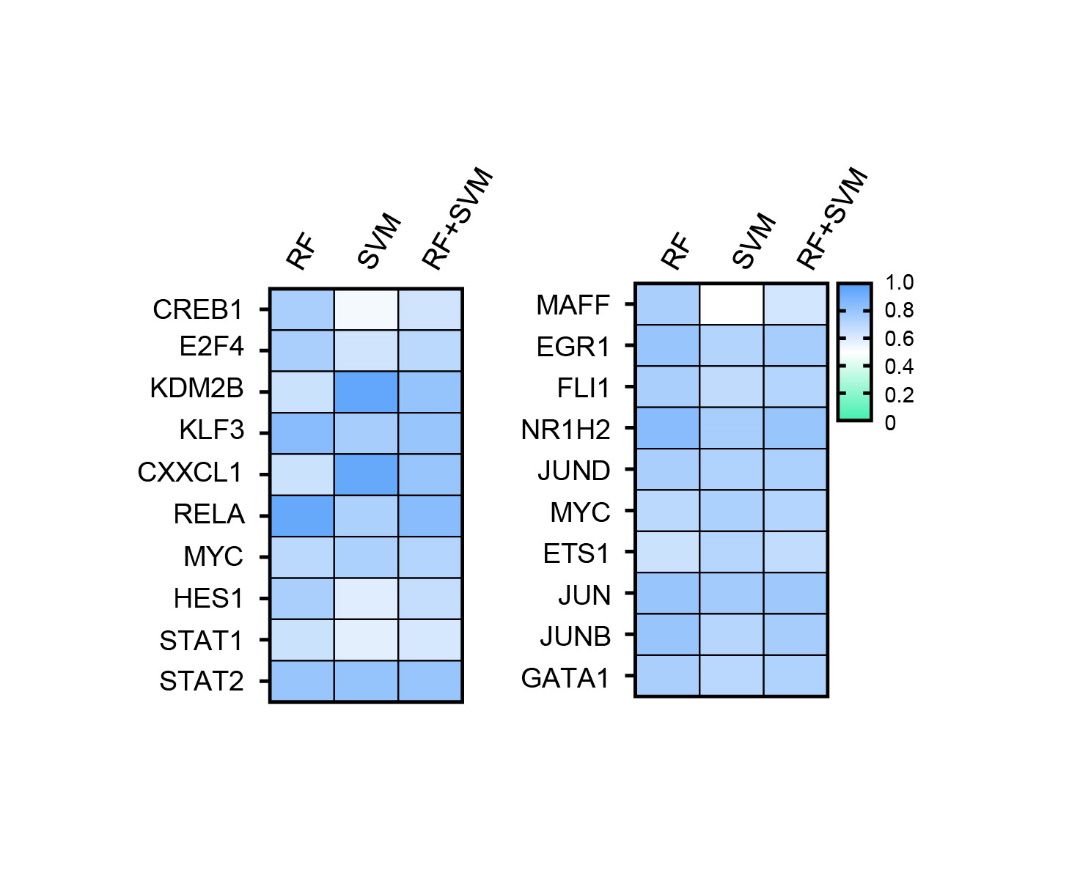


**Figure S8. The probability of** **interaction between circTmcc1 and specific transcriptional factor.** RPIseq was used to confirm the interaction between circTmcc1 and the transcription regulator. RF and SVM refer to random forest and support vector forest, respectively. The color bars show the possibility score (0–1) of interaction.


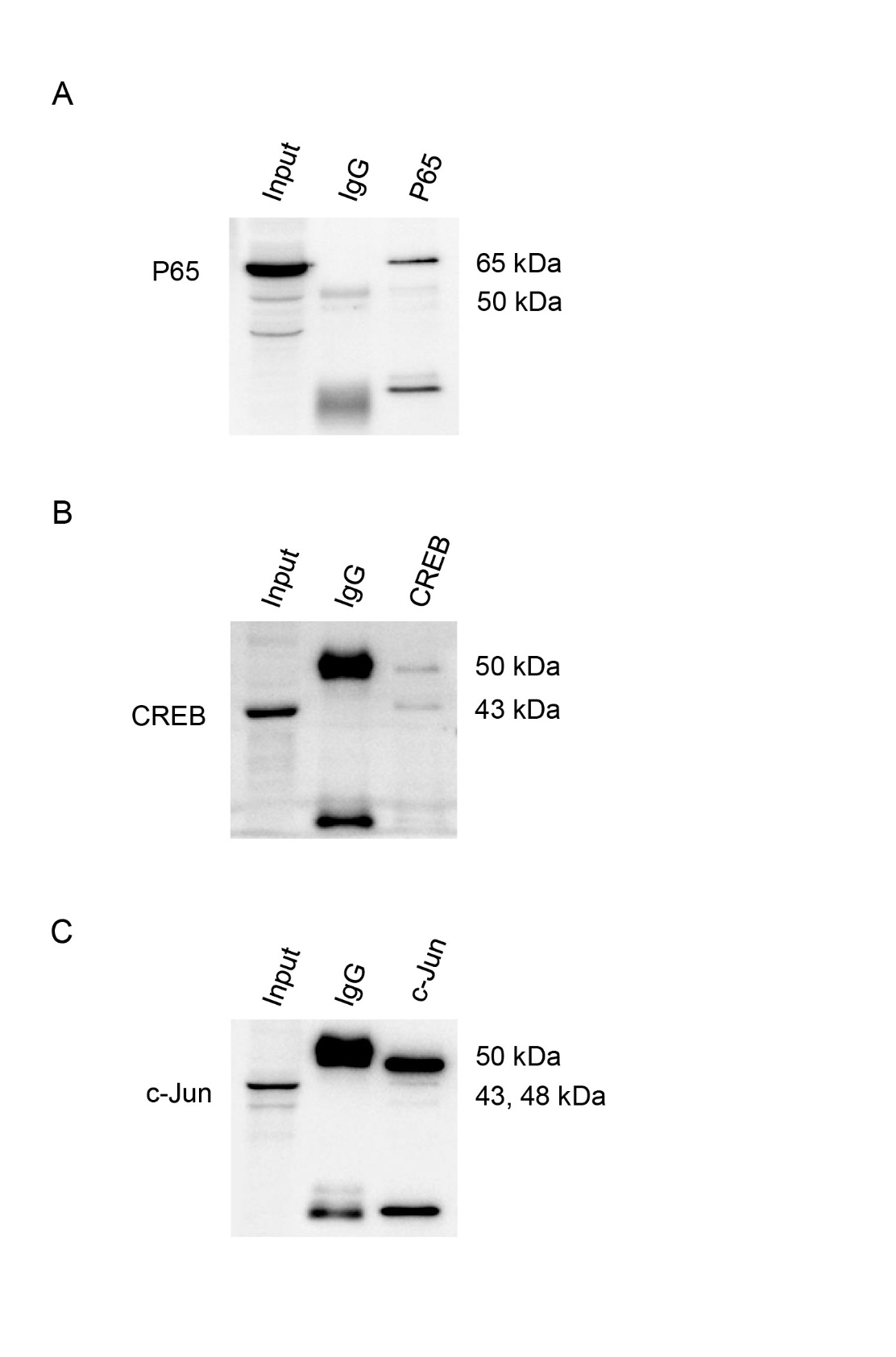


**Figure S9. RNA-binding protein immunoprecipitation (RNA-IP) of circTmcc1.** (**a**) Interaction between circTmcc1 and p65 NF-κB. (**b**) Interaction between circTmcc1 and CREB. (**c**) Interaction between circTmcc1 and c-Jun. M indicates the RNA size ladder. The expression of p65 NF-κB protein in cell lysates after immunoprecipitation with magnetic beads and antibody complexes.


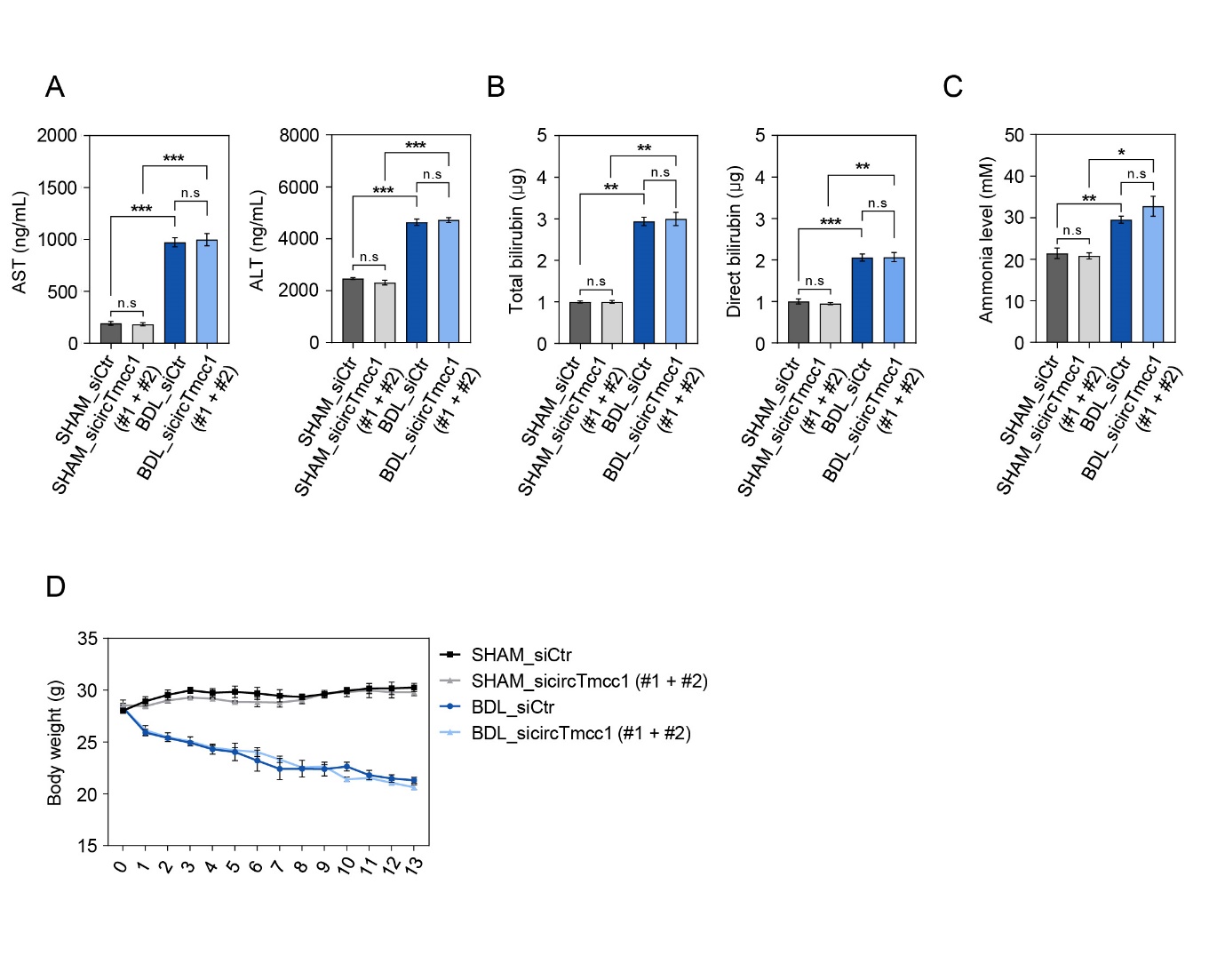


**Figure S10. Liver function-related markers changes in BDL mice plasma.** SHAM-siCtr indicates control siRNA-infused sham mice, and sham-sicircTmcc1 (#1+#2) indicates the sham mice infused with two circTmcc1 siRNAs. BDL_siCtr indicates control siRNA-infused BDL mice, whereas BDL_sicircTmcc1 (#1+#2) indicates the BDL mice infused with two circTmcc1 siRNAs. Data are described as the mean ± SEM (*n* = 3). (**a)** Measurement of ALT and AST levels in the plasma of sham and BDL mice following siRNA pump infusion. (**b)** Measurement of total and direct bilirubin levels in the plasma of both sham and BDL mice following siRNA pump infusion. (**c)** Quantification of ammonia level in the sham and BDL mice plasma following siRNA pump infusion. (**d**) Measurement of body weight of siRNA osmotic pump-infused sham and BDL mice after bile duct ligation surgery.


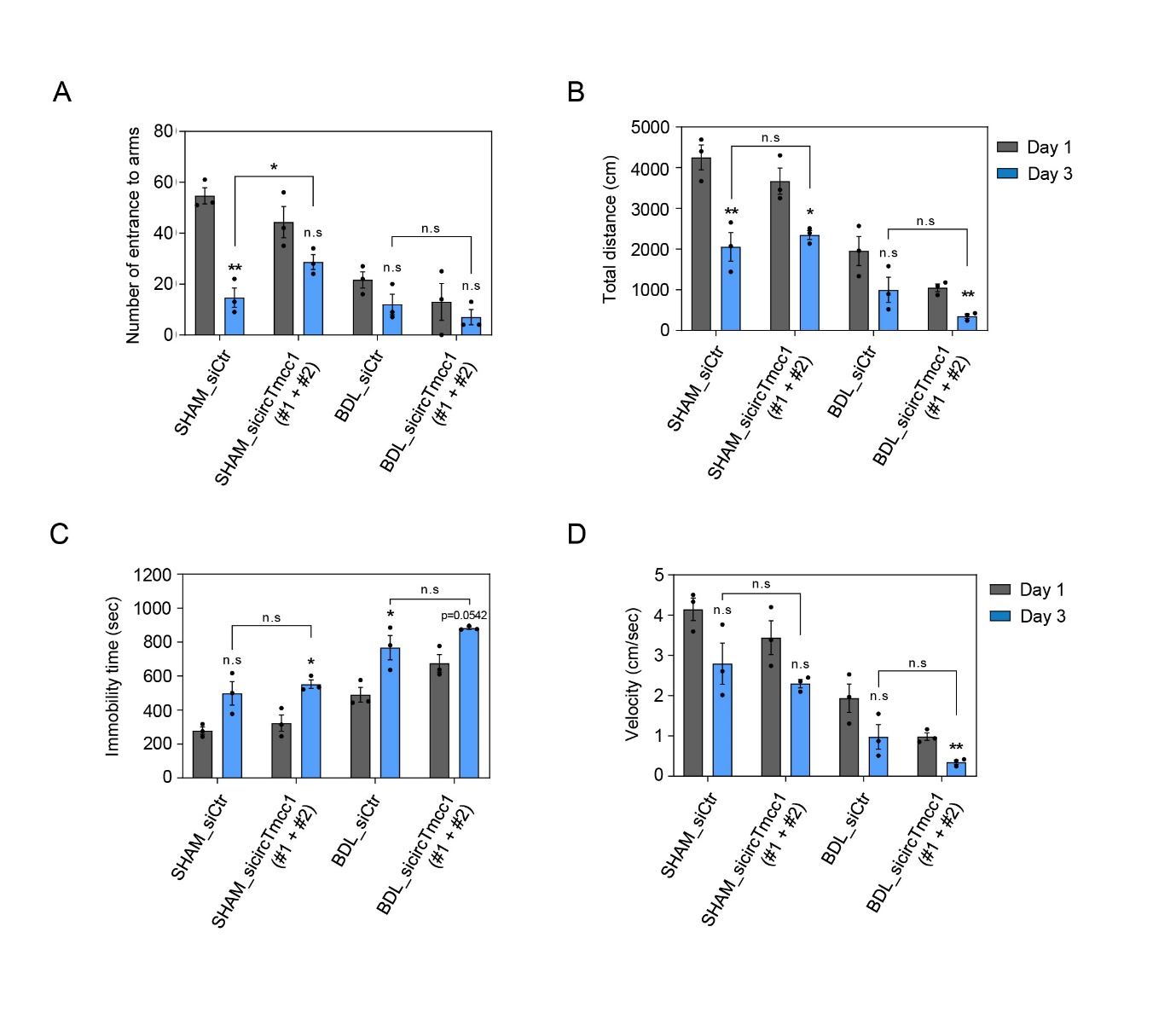


**Figure S11. The locomotor activities of brain osmotic pump-infused sham and BDL mice.** SHAM_siCtr indicates control siRNA-infused sham mice, and sham-sicircTmcc1 (#1+#2) indicates the sham mice infused with two circTmcc1 siRNAs. BDL_siCtr indicates control siRNA-infused BDL mice, and BDL_sicircTmcc1 (#1+#2) indicates the BDL mice infused with two circTmcc1 siRNAs. **(a)** The changes in the number of entrances to the arms, (**b**) the changes in the total length of mouse movement (cm), (**c**) the changes in the immobility time of mouse (seconds), and (**d**) the changes in the velocity of mouse movement (cm/s), for sham and BDL mice following pump infusion of either control siRNA or circTmcc1 siRNAs, respectively. Data were measured at days 1 and 3 during the experiment and are reported as the mean ± SEM (*n* = 3).
